# Locking mixed-lineage kinase domain-like protein in its auto-inhibited state prevents necroptosis

**DOI:** 10.1073/pnas.2017406117

**Published:** 2020-12-14

**Authors:** Martin Rübbelke, Dennis Fiegen, Margit Bauer, Florian Binder, James Hamilton, Jim King, Sven Thamm, Herbert Nar, Markus Zeeb

**Affiliations:** ^a^Medicinal Chemistry, Boehringer Ingelheim Pharma GmbH & Co. KG, 88397 Biberach an der Riss, Germany;; ^b^Bioprocess Development Biologicals, Boehringer Ingelheim Pharma GmbH & Co. KG, 88397 Biberach an der Riss, Germany;; ^c^Immunology and Respiratory Disease Research, Boehringer Ingelheim Pharmaceuticals, Inc., Ridgefield, CT 06877;; ^d^Drug Discovery Sciences, Boehringer Ingelheim Pharma GmbH & Co. KG, 88397 Biberach an der Riss, Germany

**Keywords:** necroptosis, drug discovery, NMR, X-ray

## Abstract

As an alternative pathway of controlled cell death, necroptosis can be triggered by tumor necrosis factor via the kinases RIPK1/RIPK3 and the effector protein mixed-lineage kinase domain-like protein (MLKL). Upon activation, MLKL oligomerizes and integrates into the plasma membrane via its executioner domain. Here, we present the X-ray and NMR costructures of the human MLKL executioner domain covalently bound via Cys86 to a xanthine class inhibitor. The structures reveal that the compound stabilizes the interaction between the auto-inhibitory brace helix α6 and the four-helix bundle by stacking to Phe148. An NMR-based functional assay observing the conformation of this helix showed that the F148A mutant is unresponsive to the compound, providing further evidence for the importance of this interaction. Real-time and diffusion NMR studies demonstrate that xanthine derivatives inhibit MLKL oligomerization. Finally, we show that the other well-known MLKL inhibitor Necrosulfonamide, which also covalently modifies Cys86, must employ a different mode of action.

## Significance

Necroptosis is an emergency controlled cell-death mechanism, which only may be activated in case of impaired apoptosis. Necroptosis leads to unchecked release of proinflammatory cytoplasmatic content of the cells and is thereby implicated in disease. Two covalent inhibitor classes targeting the same cysteine of the necroptosis effector protein mixed-lineage kinase domain-like protein (MLKL) are reported. Here, we unravel the mode of action of the xanthine class of inhibitors that work by stabilizing the inactive state of MLKL by an essential π–π stacking interaction. The widely used covalent tool compound Necrosulfonamide (NSA) employs a distinct mode of action.

Controlled cell death is essential for tissue homeostasis. Besides the well-studied controlled cell-death mechanism apoptosis, other pathways of controlled cell death exist. One of the best-studied alternative mechanisms is Tumor Necrosis Factor (TNF)-induced necroptosis, which depends on TNF receptor 1, Receptor-Interacting Protein Kinase 1 (RIPK1), RIPK3, and the mixed-lineage kinase domain-like protein (MLKL) (1). TNF induces necroptosis when caspase activity is reduced (2). In contrast to apoptosis, necroptosis leads to the massive release of damage-associated molecular patterns, some of which have proinflammatory activity (3). Consequently, necroptosis has been associated with several inflammatory diseases, including amyotrophic lateral sclerosis (4), chronic intestinal inflammation (5), and skin inflammation (6). Therefore, all three proteins involved in the canonical necroptosis signaling and execution cascade (RIPK1, RIPK3, and MLKL) are attractive molecular targets for therapeutic small-molecule intervention (7).

MLKL, the effector in the necroptosis signal chain, consists of two domains connected by two so-called brace helices, as described by Murphy et al. (8) for the mouse full-length protein. Structural information on human full-length MLKL is not available. However, individual domains could be characterized. The structure of the C-terminal domain of human MLKL was solved by X-ray crystallography (9) and reveals a catalytically inactive pseudokinase domain. Upon phosphorylation of the pseudokinase domain (Thr356 and Ser357) by RIPK3 (10), the activation signal is relayed to the N-terminal executioner domain. The solution structure of the latter has been solved by NMR spectroscopy by two laboratories (11, 12) and consists of a four-helix bundle (α1, α2, α3, and α5) as well as two additional α-helices (α4 and α6). The first brace helix (α6) plays an important role in the proposed plug-release mechanism (11). It postulates that phosphorylation of MLKL destabilizes the packing of the auto-inhibitory α-helix 6 to the four-helix bundle and, thereby, promotes the detachment of α-helix 6 from the four-helix bundle. The MLKL four-helix bundle is now able to oligomerize and integrates into the plasma membrane (13), which leads to permeabilization of the membrane and the breakdown of the cellular structure.

Inhibition of MLKL-induced pore formation can be achieved by different modes of action. First reports of successfully targeting the nucleotide-binding site of the pseudokinase domain (14) could not be confirmed as a specific MLKL-mediated effect due to additional effective inhibition of RIPK1 and RIPK3, which is most likely responsible for the observed antinecroptotic effect of that compound (15). Furthermore, compounds that are more selective for the MLKL pseudokinase nucleotide-binding site show no impact on necroptosis (15). In a cellular high-throughput screen (HTS) comprising ∼200,000 compounds, two different chemical series of necroptosis inhibitors targeting the N-terminal four-helix bundle were discovered (10). The first inhibitor, Necrosulfonamide (NSA), binds covalently to Cys86 located in α-helix 4 of MLKL. Its optimization turned out to be challenging, due to steep structure–activity relationships (SARs) (10, 16). However, NSA is a widely used chemical probe. Compounds of the second class identified in the same HTS, the xanthines, also covalently modify Cys86. They show tractable SARs with the most potent compound revealing a half-maximal inhibitory concentration (IC_50_) of 2 nM in a cellular necroptosis assay (17).

Here, we present the high-resolution structures of the human MLKL executioner domain in complex with a covalent inhibitor of the xanthine class solved by X-ray crystallography and in solution by NMR spectroscopy. Both complex structures revealed a π–π stacking interaction between Phe148 and the xanthine core, which turned out to be the essential element of its inhibitory mode of action. Mutational analysis and an in vitro NMR functional assay were employed to investigate the inhibitory effects of the different small molecules supporting the finding that the xanthine class and NSA act via distinct mechanisms.

## Results

**Fig. 1.**
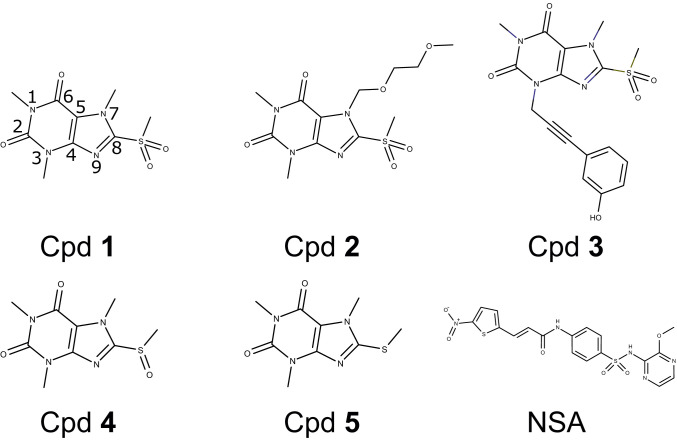
Structural formulas of covalent MLKL inhibitors. Atoms in the xanthine core of Cpd **1** are numerated.

To get a better understanding of the underlying mechanism of MLKL inhibition, we set out to determine the costructure between known inhibitors and the MLKL executioner domain. As a first step in the selection process of a suitable MLKL inhibitor for structural analysis, we confirmed the functional activity of published necroptosis inhibitors in two cellular assays. NSA (Fig. 1) shows moderate IC_50_ values in the high nanomolar to micromolar range (Table 1). Structural characterization of the NSA binding mode was unsuccessful both in soaking and cocrystallization trials. Structural analysis by NMR spectroscopy was also hampered due to significant line broadening in fingerprint spectra (see ^1^H,^15^N-correlation spectrum in Fig. 6) and consecutively insufficient quality of nuclear Overhauser enhanced spectroscopy (NOESY) spectra (*SI Appendix*, Fig. S1). The xanthine class shows nanomolar IC_50_ values in cellular necroptosis assays in FADD-deficient Jurkat and U937 cells with a significantly better ratio between activity and toxicity (Table 1, Cpd **1**+**2** [Fig. 1]). Derivatives of Cpd **1** with modified less-reactive sulfoxide (Cpd **4** [Fig. 1]) or nonreactive sulfide (Cpd **5** [Fig. 1]) moieties showed reduced or no activity. Crystallization trials of Cpd **1** and the most active xanthine (Cpd **2**) with the MLKL executioner domain did not yield suitable cocrystals amenable for X-ray crystallography. NMR measurements with Cpd **2** resulted in very-well-dispersed fingerprint spectra with major chemical-shift perturbations (CSPs) (*SI Appendix*, Fig. S2 *A* and *B*). Backbone and side-chain assignments could be achieved by employing three-dimensional (3D) triple-resonance experiments, and nuclear Overhauser effect (NOE) spectra were collected to generate intraprotein distance restraints. Unfortunately, in 3D-filtered edited NOESY spectra, intermolecular cross-peaks could only be observed for the two methyl groups attached to the xanthine core, but not for the protons of the large substitution, including the phenyl ring (*SI Appendix*, Fig. S2*C*). A reliable costructure determination was therefore not possible. Thus, we synthesized several derivatives with similar potency in the cellular necroptosis assays. Among these, Cpd **3** (Fig. 1) proved to be more suitable for structural analysis by both X-ray crystallography and NMR spectroscopy.

**Table 1.** IC_50_ values for covalent MLKL inhibitors determined in cell-based necroptosis (NEC), apoptosis (APOP), or toxicity (TOX) assays in Jurkat and U937 cells

	Jurkat NEC IC_50_, nM	Jurkat TOX IC_50_, nM	U937 NEC IC_50_, nM	U937 APOP IC_50_, nM	U937 TOX IC_50_, nM
Cpd **1**	574	56,280	312	>100,000	>100,000
Cpd **2**	69	3,835	42	>100,000	4,375
Cpd **3**	541	13,579	271	>100,000	25,462
Cpd **4**	5,235	>100,000	3,167	>100,000	>100,000
Cpd **5**	>100,000	>100,000	>100,000	>100,000	>100,000
NSA	1,399	6,197	454	>100,000	14,694

### Structures of the Complex of MLKL Executioner Domain and Cpd 3.

**Fig. 2.**
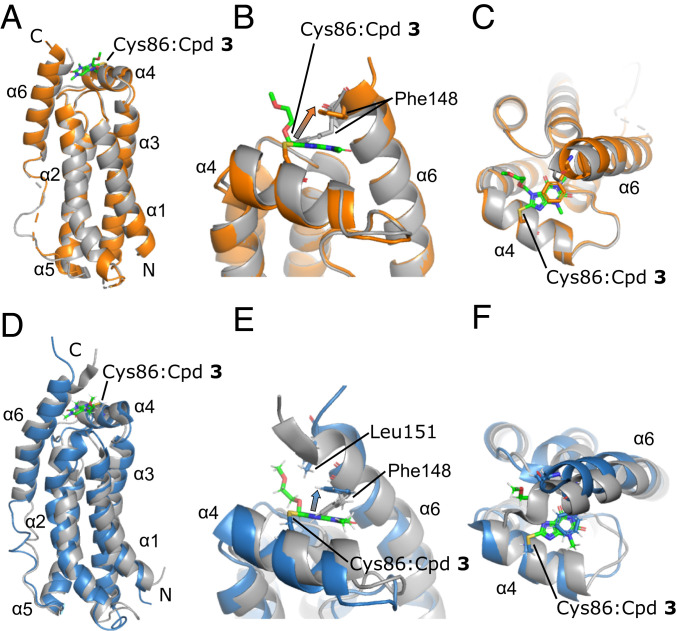
(*A*–*C*) Superimposition of the X-ray structures of unliganded MLKL executioner domain (gray; PDB ID code 6ZVO) and covalently bound to Cpd **3** (orange; PDB ID code 6ZZ1). (*D*–*F*) Superposition of the NMR structures of unliganded MLKL executioner domain (gray; PDB ID code 6ZLE) and covalently bound Cpd **3** (blue; PDB ID code 6ZPR). The compound is presented as sticks and colored in green. Key interacting residues are shown as sticks. The conformational change of Phe148 located in the autoinhibitory α-helix 6 is indicated by arrows. N and C termini are indicated.

First, we solved the structure of the unliganded MLKL executioner domain by X-ray crystallography (*SI Appendix*, Table S2) and NMR spectroscopy to have a basis for detailed analysis of structural changes induced by small-molecule binding. Both structures of the unliganded four-helix bundle were highly similar (rmsd value 2.1 Å) and showed only minor differences from the two published NMR solution structures [NMR structure has an rmsd of 2.4 Å to 2MSV (11) and an rmsd of 2.5 Å to 6D74 (12)] (*SI Appendix*, Fig. S3). The four-helix bundle was formed by α-helices 1, 2, 3, and 5 of MLKL with a short interjacent α-helix 4 on top. The flexible linker between α-helices 5 and 6 (residues 120 to 130) was not fully visible in the X-ray structures due to inherent flexibility. The subsequent α-helix 6 packed with multiple contacts, against the four-helix bundle (Fig. 2 *A* and *D*). The ^15^N NMR relaxation properties (*T*_1_, *T*_2_, heteronuclear NOE) of α-helix 6 were comparable to the helices forming the four-helix bundle, which indicated a rather rigid packing against the four-helix bundle. The calculated molecular correlation time (τ_C_ = 16 ns) for the rigid parts of MLKL was higher than expected for a spherical protein of its size (*SI Appendix*, Fig. S4). However, this may be explained by the elongated shape of MLKL, resulting in an anisotropic rotational diffusion. Interestingly, α-helix 4 showed a reduced molecular correlation time (τ_C_ = 12 ns), which also can be explained by the anisotropy, since helix 4 is oriented perpendicular to the four-helix bundle, and the N–H bond vectors therefore experience different molecular rotation.

Next, we determined the protein–ligand costructure with the newly synthesized xanthine Cpd **3** (Fig. 2). In filtered edited 3D NOESY NMR spectra, 41 intermolecular NOEs between MLKL and Cpd **3** (*SI Appendix*, Table S1 and Fig. S2*C*) could be assigned unambiguously and lead to a well-converged binding pose of the ligand in the structural NMR bundle (*SI Appendix*, Fig. S5). The binding pose of Cpd **3** was very similar in the NMR and the X-ray structures of the MLKL:Cpd **3** complex. Overall structural changes upon complex formation were minor, and the overall fold of MLKL was preserved. Backbone ^15^N NMR relaxation data of the complex were also very similar to unbound MLKL, and even the dynamics of the α-helix 4, where Cys86 is located, did not differ (*SI Appendix*, Fig. S4). The most profound structural change induced by covalent modification of Cys86 was the alteration of the conformation of Phe148 (arrows in Fig. 2 *B* and *E*). This resulted in a stacking interaction with the aromatic ring of the xanthine core, therefore creating an additional contact between α-helix 6 and the four-helix bundle. Minor differences between the NMR and X-ray costructure were most likely due to variations in construct lengths (residues 1 to 154 in the NMR and 1 to 150 in the X-ray structure, respectively). For example, intermolecular NOEs were detected from the Leu151 methyl groups to the ethylene glycol moiety, indicative of additional van der Waals contacts of Cpd **3** to the C-terminal brace helix (α-helix 6).

### NMR-Based Analysis of Additional Compounds.

Covalent binding of Cpd **1** and **2** to the MLKL executioner domain induced very similar changes in chemical shift observed in ^1^H,^15^N-correlation spectra compared to the binding of Cpd **3** (*SI Appendix*, Fig. S6), indicating that the binding mode of the three compounds is very similar. Nevertheless comparing the spectra of the MLKL executioner domain bound to Cpd **1**, which is decorated with methyl groups on all three nitrogen atoms of the xanthine core (N1, N3, and N7), with spectra bound to Cpd **3**, which feature an ethylene glycol moiety in position N7, showed small CSPs evenly spread around the xanthine binding site. In contrast, comparing spectra of the executioner domain bound to Cpd **1** and **2**, which features an alkyne bridge to a phenyl ring in position N3 instead of a methyl group, the differences were focused within the C terminus of α-helix 6. This is consistent with a model in which the additional phenyl group is pointing toward the C-terminal residues in α-helix 6 (*SI Appendix*, Fig. S7). The modeled pose was consistent with the observed lack of intermolecular NOEs between the phenyl ring in Cpd **2** and the MLKL executioner domain, since there was plenty of room for the phenyl ring to move and no specific interaction holding it in place.

### Covalent Xanthine Inhibitors Trap the MLKL Executioner Domain in the Auto-Inhibited State.

**Fig. 3.**
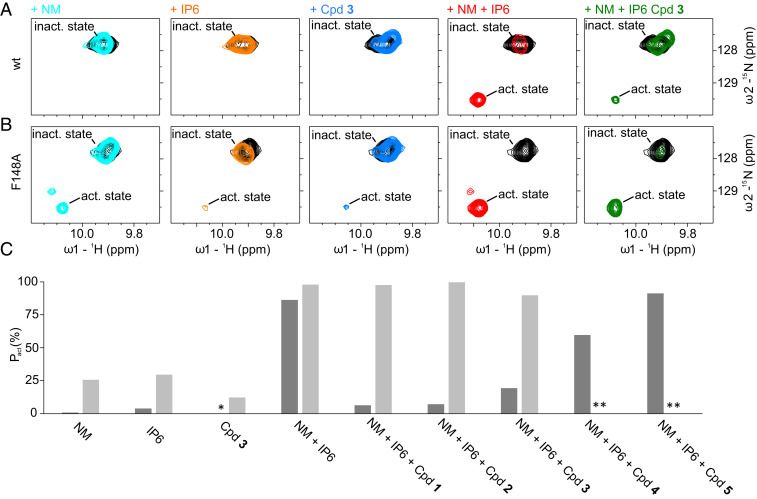
(*A* and *B*) The region of the NMR ^1^H,^15^N-correlation spectra of the MLKL executioner domain (protein concentration: 80 µM) containing the cross-peaks of Trp133 Nε1–Hε1 in the auto-inhibited state where α-helix 6 is attached to the four-helix bundle (inact. state) and in the active, detached state (act. state) are shown. The unliganded state is depicted in black, with 12 mM NM in cyan; 500 µM IP6 in orange; 500 µM Cpd **3** in blue; 12 mM NM and 500 µM IP6 in red; and 12 mM NM, 500 µM IP6, and 500 µM Cpd **3** in green. The spectra depicted in *A* are measured with the WT protein and in *B* with the F148A variant. (*C*) Population of the active conformation (p_act_) in percent where α-helix 6 is detached from the four-helix bundle calculated from integrals of Trp133 Nε1–Hε1 cross-peaks in the active and inactive states. Dark gray bars correspond to the WT, while light gray bars correspond to the F148A variant. The asterisk means that no active conformation could be detected. Double asterisks indicate data not determined.

The detachment of α-helix 6 has been shown to be a key step in activation of MLKL and membrane integration of the MLKL executioner domain (12). To test the significance of the additional interaction between α-helix 6 and the four-helix bundle provided by the xanthine compounds stacking to the phenyl ring of Phe148, we used a described functional NMR assay (12). Binding of phytic acid (IP6) and the detergent β-d-nonylmaltoside (NM) led to the detachment of α-helix 6, which can be monitored in ^1^H,^15^N-correlation spectra by the altered chemical shift of the Hε1–Nε1 correlation of Trp133 and the backbone N–H correlation of Ala141 (12). The ^1^H,^15^N-correlation spectrum of the unliganded MLKL four-helix bundle showed only one peak for the Hε1–Nε1 correlation of Trp133 (Fig. 3), consistent with its side chain packed between α-helices 2 and 5 and, thereby, anchoring α-helix 6 to the four-helix bundle. Adding 12 mM NM or 500 µM IP6 led to the appearance of a second very weak peak reporting on the detached α-helix 6 with a population of the activated state (*p*_act_) of 1% and 4%, respectively (Fig. 3). Cpd **3** in contrast, did not induce the active state (Fig. 3). Combining 12 mM NM and 500 µM IP6 induced a dramatic increase of the population of the detached state (*p*_act_ 86%). The additional peak reporting for the active state of Ala141 was also clearly visible and showed comparable results. Addition of 500 µM Cpd **3** shifted the population back to the auto-inhibited state of α-helix 6 (*p*_act_ 19%), suggesting that the stabilization by the covalent modification protects MLKL from activation (Fig. 3). Comparable results were observed for two additional xanthine compounds (Cpd **1** and **2**), for which an even less activated MLKL executioner domain was present after compound addition (Fig. 3*C*). Reducing the warhead reactivity (Cpd **4**) or replacing it by a nonreactive moiety (Cpd **5**) significantly reduced or abolished the inhibitory effect (Fig. 3*C*), which agrees very well with the results of the cellular necroptosis assay (Table 1). To further validate the importance of the π–π stacking interaction between the xanthine and Phe148 in α-helix 6, we carried out the functional NMR assay with the F148A-mutated variant of MLKL. The removal of the phenyl group induced significant CSPs localized in close proximity to the mutation site, due to the removal of ring currents and, therefore, strong electronic changes. The mutation did not alter the backbone secondary chemical shifts or ^15^N-relaxation parameters significantly (*SI Appendix*, Fig. S8), indicating that MLKL F148A adopts a very similar overall structure with comparable backbone dynamics. The most pronounced difference was that residues comprising α-helix 4 in the wild-type (WT) protein showed line broadening to the point that corresponding peaks were not detectable in triple-resonance experiments, and, therefore, secure assignment and analysis of the secondary structure of these residues was not possible. MLKL F148A was more sensitive to the addition of 12 mM NM, 500 µM IP6, or the combination thereof. In the spectra in the presence of NM and the combination of NM and IP6, a third Trp133 peak appeared, most likely reporting on a state similar to the active state. The addition of Cpd **3** alone led to a small population of the active state (Fig. 3). In contrast to WT MLKL, addition of the xanthine compounds in the presence of 12 mM NM and 500 µM IP6 did not prevent the detachment of α-helix 6 from the four-helix bundle in the F148A mutant (*p*_act_ 90% for Cpd **3**). The resulting activation is comparable to WT in the absence of Cpd **3** (Fig. 3). Thus, the stacking interaction of the xanthine core to Phe148 is vital for the inhibitory activity of these MLKL inhibitors.

### Xanthines Decelerate Oligomerization of the MLKL Executioner Domain.

**Fig. 4.**
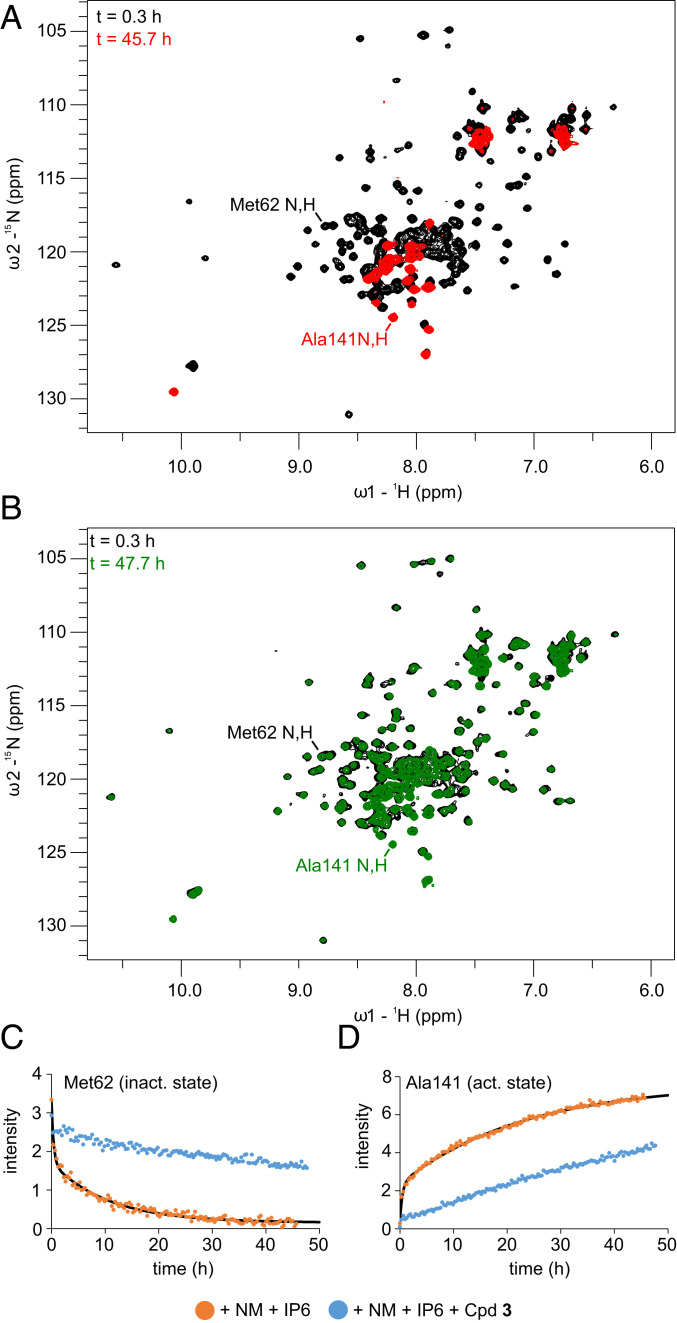
(*A*) The ^1^H,^15^N-correlation spectra of MLKL executioner domain in the presence of 12 mM NM and 50 µM IP6 directly after adding 50 µM IP6 (black) and after 45.7 h at 298 K (red). (*B*) The ^1^H,^15^N-correlation spectra of the MLKL executioner domain in the presence of 12 mM NM, 50 µM IP6, and 500 µM Cpd **3** directly after adding 50 µM IP6 (black) and after 47.7 h at 298 K (green). Assignments of selected peaks are indicated. (*C* and *D*) Peak intensity from the time course of ^1^H,^15^N-correlation spectra for Met62 (*C*) and for the activated, α-helix 6 detached state of Ala141 (*D*) for the MLKL executioner domain under the condition described in *A* (orange) and with additionally 500 µM Cpd **3** (blue). Solid black lines indicate biexponential fitting curves for the time course in presence of NM and IP6. Act., active; inact, inactive.

While MLKL was stable in the presence of either 500 µM IP6 or 12 mM NM (*SI Appendix*, Fig. S13), dispersed peaks were disappearing over time in the presence of the combination of the two. Monitoring this decay by real-time NMR, a series of ^1^H,^15^N-correlation spectra were recorded in the presence of 12 mM NM and 50 µM IP6. The latter concentration was reduced to slow down the process to enable real-time NMR detection, because at 500 µM IP6 concentration, 86% activation could be observed in the functional assay in the ^1^H,^15^N-correlation spectrum measured directly after sample preparation. The loss of peak intensity can be fitted with a biexponential decay with a fast time constant (T_f_) of 0.35 ± 0.07 h and a slow time constant (*T*_s_) of 10.0 ± 1.0 h. The time constants were comparable for all six α-helices of MLKL, indicating a simultaneous cooperative conformational change or transition to an oligomeric state of the protein (Fig. 4 *C* and *D* and *SI Appendix*, Table S3). In addition to the known peaks reporting on the active state with α-helix 6 being detached (Ala141 N,H and Trp133 Nε1,Hε1), peaks in the random coil region of the spectrum appeared (Fig. 4*A*). At the end of the time course, much fewer peaks were present in the spectrum compared to the guanidinium chloride-induced fully unfolded MLKL (*SI Appendix*, Fig. S9*A*), suggesting that only a small part of the protein was unfolded and remained detectable. Backbone assignment of these peaks revealed that they belonged to residues 129 to 154, comprising the residues of α-helix 6 in the native inactive state (*SI Appendix*, Fig. S9*B*). Secondary chemical shifts confirmed that α-helix 6 becomes largely unfolded and has, according to ^15^N-relaxation data, increased mobility (τ_C_ = 10 ns) and reduced rigidity (heteronuclear NOE around zero) in the activated state (*SI Appendix*, Fig. S10). Only residues 142 to 147 showed small positive secondary chemical shifts, slightly less reduced τ_C_ values, and lower heteronuclear NOEs, indicating a small α-helical propensity. The uniform disappearance of the dispersed peaks of the four-helix bundle in ^1^H,^15^N correlation spectra points toward an oligomerization or aggregation of the four-helix bundle in the presence of IP6 and NM, while the high flexibility of the now-unfolded α-helix 6 indicates that it tumbles freely in solution. To characterize the secondary structure of the NMR invisible oligomerized state, far-ultraviolet circular dichroism (CD) spectra of the MLKL executioner domain were recorded, which showed the characteristic pattern of an α-helical protein (*SI Appendix*, Fig. S11). The slightly reduced molar ellipticity compared to monomeric MLKL can be attributed to the unfolding of α-helix 6.

Since the xanthine compounds were able to keep the MLKL executioner domain in the inactive state, a time course in the presence of 50 µM IP6, 12 mM NM, and 500 µM Cpd **3** was recorded. Under these conditions, a significantly decelerated decay of the dispersed signals was observed. After 48 h, these signals decayed only to ∼70% intensity. Cross-peaks reporting on the unfolded, detached α-helix 6 likewise appeared more slowly in the presence of Cpd **3** (Fig. 4 *B*–*D*).

### Xanthines Keep the MLKL Executioner Domain in a Monomeric State.

**Fig. 5.**
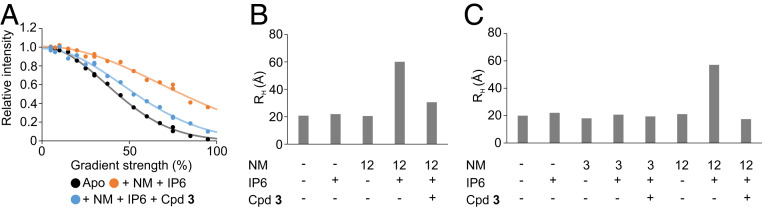
(*A*) Integral of amide region in ^15^N-edited NMR diffusion experiments plotted against the used gradient strength. Black dots depict data for the MLKL unliganded executioner domain. Orange dots depict data for the protein in the presence of 12 mM NM and 50 µM IP6, and blue dots data for the protein in the presence of 12 mM NM, 50 µM IP6, and 500 µM Cpd **3** collected succeeding the time course presented in Fig. 4. (*B*) Hydrodynamic radii for the MLKL executioner domain extracted from ^15^N-edited NMR diffusion experiments (raw data partly depicted in *A*) under the influence of NM, IP6, and Cpd **3**. The NM concentration is given in millimolar. Presence of 50 µM IP6 and 500 µM Cpd **3** is indicated by plus signs. (*C*) Hydrodynamic radii for the MLKL executioner domain under indicated conditions extracted from regular, not filtered, NMR diffusion experiments. The NM concentration is given in millimolar. The presence of 500 µM IP6 or 500 µM Cpd **3** is indicated by plus signs.

To further characterize the oligomerized MLKL executioner domain, NMR diffusion experiments were conducted directly succeeding the end of the real-time NMR series (orange dots in Fig. 5*A*). This was compared with a fresh sample of unliganded MLKL executioner domain (black dots in Fig. 5*A*). The unliganded MLKL executioner domain had a hydrodynamic radius of 19 Å (Fig. 5*B*), which is consistent with a monomer. In contrast, the oligomerized MLKL executioner domain under the influence of 12 mM NM and 50 µM IP6 had a strongly increased hydrodynamic radius of 60 Å, which indicates oligomerization. The NMR diffusion experiments show that the hydrodynamic radius of the MLKL executioner domain in complex with Cpd **3** in the presence of NM and IP6 was 30 Å and, therefore, 30 Å smaller than in the absence of Cpd **3**. Taken together, the NMR data suggest that the xanthine class of compounds slows oligomerization of the MLKL executioner domain by keeping the protein in the inactive, α-helix 6 attached monomeric conformational state.

In a next step, we conducted NMR diffusion experiments with NM concentrations above and below the critical micelle concentration of 6 mM, to find out if the increased hydrodynamic radius of the MLKL executioner domain is dependent on the presence of micelles. NM dissolved in buffer without protein had a higher hydrodynamic radius at 12 mM than at 3 mM, indicating the formation of micelles at higher NM concentrations. The presence of 500 µM IP6 and 500 µM Cpd **3** had no influence on the hydrodynamic radius of NM (*SI Appendix*, Fig. S12). While the MLKL executioner domain displayed a hydrodynamic radius of 57 Å in the presence of 500 µM IP6 and NM micelles (12 mM) after about 2 d of incubation, it stayed monomeric in the absence of micelles (3 mM NM) (Fig. 5C). Therefore, the oligomerization depends on micelles, and the observed species is most likely a micelle-integrated oligomeric MLKL executioner domain. In the presence of NM micelles (12 mM NM) and 500 µM IP6, after 2 d of incubation, a stronger protective effect of 500 µM Cpd **3** was observed compared to the NMR time course with only 50 µM IP6 present, resulting in a completely monomeric MLKL executioner domain. The differences between the experiments with substoichiometric IP6 concentration (50 µM) and excess of IP6 (500 µM) in the presence of the compound can probably be explained by unspecific aggregation in the substoichiometric case, which led to an apparent higher hydrodynamic radius of the inhibited MLKL.

### NSA Employs a Different Mode of Action.

**Fig. 6.**
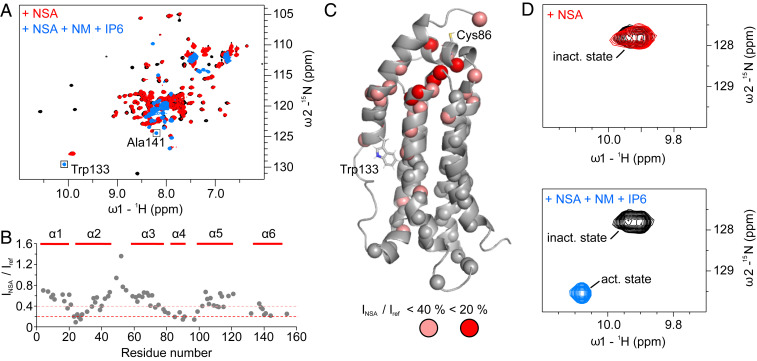
(*A*) NMR ^1^H,^15^N-correlation spectrum of 80 µM unliganded MLKL executioner domain (black), in complex with NSA (red) at a concentration of 200 µM, which is sufficient to saturate the protein, and additionally in the presence of 12 mM NM and 500 µM IP6 (blue). (*B*) Ratio of the peak intensities of the MLKL executioner domain:NSA complex over the unliganded protein extracted from spectra shown in *A*. (*C*) NMR structure of the MLKL executioner domain. Spheres indicate residues for which the peak height in the unliganded and the NSA bound state could be compared. Salmon spheres indicate residues that have peak intensities smaller than 40%, and red spheres residues with peak intensities smaller than 20% in the NSA bound state. (*D*) Regions of the spectrum the ^1^H,^15^N-correlation spectra containing the Trp133 Nε1–Hε1 cross-peaks in the auto-inhibited state where α-helix 6 is attached to the four-helix bundle (inact. state) and in the active state where it is detached (act. state).

Besides the xanthine class, we also investigated NSA, the first identified MLKL inhibitor, which also covalently modifies Cys86, and addressed the question of if NSA is able to prevent the detachment of α-helix 6 in the presence of NM and IP6. In contrast to the xanthine class, NSA induced no CSP, but, rather, significant and specific line broadening of the MLKL executioner domain cross-peaks in the ^1^H,^15^N-correlation spectrum (Fig. 6*A*). The residues Tyr23, Cys24, Lys26, Gln27, Ile-85, Thr90, Ala91, and Leu97 showed strong line broadening with peak intensities smaller than 20% of the unliganded protein. These and additional residues in α-helices 2, 4, and 6 experienced a line-broadening cluster around Cys86, indicating a flexible binding pose of NSA. This is supported by the observation that only a few intermolecular NOEs (consistent to originate from residues Leu89 and Thr90) could be detected (*SI Appendix*, Fig. S1). It suggests that the covalent bond anchors the inhibitor to Cys86, and the remaining parts hardly interact with the MLKL executioner domain. To further address the inhibitory mechanism of NSA, we performed the NMR-based functional assay. The signature cross-peak of Trp133 Hε1–Nε1 showed no CSP upon binding of NSA to the executioner domain, indicating that α-helix 6 remains in the attached auto-inhibitory state. Addition of 12 mM NM and 500 µM IP6 to the MLKL:NSA complex led to a complete population shift to the active α-helix detached state. Hence, NSA is not able to prevent the detachment of the brace α-helix 6 under these conditions, and, therefore NSA must have a different mechanism of action compared to the xanthine class of inhibitors.

## Discussion

MLKL is a key player in the necroptosis pathway, encompassing a C-terminal regulatory and an N-terminal executioner domain. It gets activated through phosphorylation of Thr357 and Ser358 in its pseudokinase domain by RIP3 kinase (10) and subsequently oligomerizes via its executioner domain and inserts into the membrane (13). To this end, displacement of the auto-inhibitory brace helix (α-helix 6) from the four-helix bundle is required, which depends on a specific composition of phospholipids in the membrane (12, 18). The auto-inhibitory role of α-helix 6 has been demonstrated by mutations of anchoring residues Trp133 and Asp137 that weaken the packing of α-helix 6 to the four-helix bundle and increase its activity in necroptosis (12). Furthermore, the addition of IP6 and the detergent NM leads to detachment of the auto-inhibitory α-helix 6, its unfolding, and oligomerization of the four-helix bundle, which is a requirement for its membrane association (19). Here, we demonstrate that the process of oligomerization already takes place with substoichiometric amounts of IP6 (50 µM IP6 and 300 µM MLKL executioner domain) and can be described by a biexponential fit with a fast (0.35 h) and a slow (10.0 h) time constant. Furthermore, we demonstrate that the first brace helix (α-helix 6) unfolds and gets highly flexible under these conditions, which matches findings by Quarato et al. (19). In NMR diffusion experiments, we find that the oligomerization of the MLKL executioner domain in response to NM and IP6 is dependent on the presence of micelles. While the MLKL executioner domain stays monomeric in the presence of IP6 and NM below the critical micelle concentration, the hydrodynamic radius increases drastically in the presence of IP6 and NM above the critical micelle concentration. Furthermore, we demonstrate that the oligomerized form of MLKL stays α-helical. MLKL has been reported to form trimers (20), tetramers (21), or octamers (22) as active components that integrate into the membrane. The theoretical hydrodynamic radii of a trimer, tetramer, and an octamer of the executioner domain can be estimated by an empirical formula postulated by Wilkins et al. (23) to be 25, 31, and 38 Å, respectively, while two octamers would have an estimated hydrodynamic radius of 46 Å. The contribution of the micelle is difficult to estimate, since it might change size upon MLKL integration. We observe a hydrodynamic radius for the MLKL executioner domain in the presence of NM micelles of 60 Å in NMR diffusion experiments. This high hydrodynamic radius of the MLKL executioner domain in our diffusion experiments can only be explained by more than one higher-order oligomer (e.g., tetramers or octamers) integrated into the micelle. A better estimation of the oligomeric state of the MLKL executioner domain is hampered by the lack of structural information of MLKL inserted into micelles or the plasma membrane.

Necroptosis could, in principle, be inhibited either by tackling the pseudokinase domain or the executioner domain of MLKL (7). Currently, two classes of compounds are reported to inhibit MLKL by covalently binding to Cys86 in the short α-helix 4 located on top of the four-helix bundle, which comprises α-helices 1, 2, 3, and 5. Here, we report that the xanthine class of inhibitors acts by stabilizing the packing of α-helix 6 against the four-helix bundle through the formation of a stabilizing π–π-stacking interaction between Phe148 located in α-helix 6 and the xanthine core. Destabilization of packing of α-helix 6 against the four-helix bundle leads to more active MLKL in necroptosis. This was shown previously by using mutagenesis of the anchoring residue D137 (12). Conversely, stabilizing the packing of the auto-inhibitory α-helix 6 against the four-helix bundle by additional contacts provided by the compound is therefore a viable mode of action for MLKL necroptosis inhibitors. The stronger interaction decelerates oligomerization of the executioner domain and its integration into micelles, as shown here by real-time NMR and NMR diffusion experiments. Mutation of Phe148 to alanine leads to loss of the stabilization of the auto-inhibited state of MLKL by xanthine class compounds, confirming the key role of the observed π–π-stacking interaction for the activity of the xanthine class. The importance of the covalent mode of action of the xanthine class is demonstrated by the partial or complete inactivity of xanthine compounds with less or nonreactive sulfoxide or sulfide warheads, instead of the highly reactive sulfone warhead in cellular assays and the functional NMR assay. This is in agreement with findings that activity of compound Cpd **1** and Cpd **2** is hampered in MLKL knockout cells transfected with C86S-mutated MLKL (17).

This specificity to Cys86 was not demonstrated explicitly for Cpd **3**. However, due to the high structural similarity to Cpd **1** and Cpd **2**, the virtually identical behavior in the NMR functional assay, and the covalent binding to Cys86 observed in the complex structures, we assume that the inhibitory effect of Cpd **3** in cells originates solely from covalent binding to Cys86 of MLKL.

The other well-known inhibitor NSA, which also binds covalently to Cys86, is not able to stabilize the interaction between α-helix 6 and the four-helix bundle in the context of the MLKL executioner domain, as shown by the NMR functional assay. NSA therefore seems to work independently of the auto-inhibitory first brace helix. In contrast to the xanthine class of compounds that introduces well-defined changes in chemical shift in ^1^H,^15^N NMR correlation spectra upon binding to Cys86, NSA instead induces severe line broadening of peaks corresponding to residues proximal to the covalent modification site. In addition, only a few weak intermolecular NOE cross-peaks could be detected, indicating that NSA does not have a well-defined binding pose in the context of the N-terminal executioner domain alone. Using molecular-dynamics simulations with a full-length model of human MLKL with covalently bound NSA, Bansal et al. (24) show that NSA is able to lock the domain–domain orientation between the four-helix bundle and the pseudokinase domain in one inactive orientation. According to the molecular-dynamics simulations, NSA forms, besides the covalent bond to Cys86, a π-cation interaction with Lys157, which is located in the second brace helix subsequent to α-helix 6. Furthermore, displacement of Lys157 by NSA leads to the formation of several new interactions between the pseudokinase domain, the brace helix, and the four-helix bundle. In contrast to the stabilization of the packing of α-helix 6 against the four-helix bundle observed for the xanthine compounds, the simulation predicts that NSA reduces the α-helical content of the brace helices (24). The proposed mechanism for the activity of NSA fits well with the observed lack of a rigid binding pose in the context of the MLKL executioner domain, since the construct used for the structures and NMR measurements comprises the four-helix bundle, including the first brace helix (α-helix 6), which lacks most of the proposed interaction sites of NSA. In a similar fashion, the phenyl group of Cpd **2**, the xanthine with the highest cellular activity, might make additional contacts to the second brace helix or the pseudokinase domain in the context of full-length MLKL. We cannot observe any NOE signals to this part of Cpd **2**, since when bound to the N-terminal executioner domain, it probably moves freely in the solvent.

In contrast to the regular full-length MLKL1, MLKL exists in a second isoform (MLKL2) that is generated by alternative splicing. MLKL2 contains the executioner domain, the two brace helices, and the C terminus of MLKL, but lacks most of the regulatory pseudokinase domain. MLKL2 is more active than MLKL1 in triggering necroptosis in cellular assays (25), but should be amenable to inhibition by the xanthine class, since all relevant residues are included in the shorter isoform.

In conclusion, we find that the two known classes of MLKL inhibitors, NSA and the xanthine class, have different modes of action, even though they both bind covalently to Cys86 within the N-terminal executioner domain. While NSA is reported to lock the executioner and the pseudokinase domain in an inactive domain–domain orientation, the xanthine class strengthens the inhibitory effect of α-helix 6 by introducing the essential π–π stacking to Phe148.

## Materials and Methods

### Cellular Assay.

For the Jurkat FADD^−/−^ Necroptosis assay, compounds were serially diluted in 100% dimethyl sulfoxide (DMSO) (Sigma-Aldrich catalog no. 41641), and 250 nL was transferred to a sterile 384-well microtiter plate (PerkinElmer catalog no. 6007688) via Echo liquid handler (LABCYTE). Five thousand Jurkat FADD ^−/−^ cells (ATCC catalog no. CRL-2572) in 20 µL of modified Roswell Park Memorial Institute medium (Gibco catalog no. 10491), 10% fetal calf serum (Gibco catalog no. 100500), 1x Anti/Anti (Gibco catalog no. 15240-062), 1x l-Glutamax (Gibco catalog no. 35050-087), and 1% PenStrep (SIGMA catalog no. P4333) were seeded into each well of the prepared assay plate with compound. Cells and compounds were incubated for 30 min at 37 °C (humidified incubator with 5% CO_2_). After incubation, 5 µL of recombinant human TNFα (huTNFα) (Biotrend catalog no. cyt-223; 10 ng/mL final concentration [f.c.]) was added to the cells, followed by an incubation of 24 h at 37 °C. Following the incubation, the experimental plates were equilibrated to room temperature (30 min) before 25 µL of Cell Titer Glo Luminescent Cell Viability Assay System reagent (Promega catalog no. G7571) was added to the plate wells, incubated at room temperature for ∼10 min. After incubation, the samples were analyzed for luminescence by using an Envision reader (PerkinElmer).

Each assay plate contained wells with “high” and “low” controls. High controls (100% CTL) were defined as cells treated with DMSO, and low controls (0% CTL) were defined as cells treated with 10 ng/mL recombinant huTNFα. IC_50_ values were calculated based on the percentage of control values using a four-parameter-fitting model.

MLKL antagonists were also evaluated in a necroptosis assay with U937 cells (26) (ATCC catalog no. CRL-1593.2). The assay procedure with U937 cells was as described for the Jurakt FADD^−/−^ necroptosis assay with the following modifications: 8,000 cells were seeded per well, and, in addition to the recombinant huTNFα, cells were also incubated in the presence of 10 µM (f.c.) carbobenzoxy-valyl-alanyl-aspartyl-[*O*-methyl]-fluoromethylketone (zVAD-fmk) (Enzo Life Sciences catalog no. ALX-260-020). Low controls (0% CTL) were defined as cells treated with 100 ng/mL recombinant huTNFα plus 10 µM zVAD-fmk.

Furthermore, MLKL antagonists were tested in a U937 apoptosis assay in the presence of 3 µM (f.c.) NSA (Tocris catalog no. 5025) and 100 ng/mL (f.c.) recombinant huTNFα in the same way as described for the U937 necroptosis assay. Low controls (0% CTL) were defined as cells treated with 100 ng/mL recombinant huTNFα plus 3 µM NSA.

MLKL antagonists were tested in cytotox assays by using Jurkat FADD^−/−^ and U937 cells according to the assay procedures described for the necroptosis assays, but without treatment with recombinant huTNFα and/or zVAD-fmk. High controls (100% CTL, no effect) were defined as cells treated with DMSO, and low controls (0% CTL) were defined as medium without cells (0% CTL, maximal effect).

All cellular assays were done for Cpd **1**, Cpd **2**, Cpd **3**, Cpd **4**, and Cpd **5** in duplicate and in quadruplet for NSA.

### Chemical Synthesis of Compounds.

Cpd **1**, **2**, **4**, and **5** were prepared according to the literature (17). NSA was prepared according to the literature (16). Cpd **3** was synthetized as follows: To a stirred solution of 8‐methanesulfonyl‐1,3‐dimethyl‐2,3,6,7‐tetrahydro‐1H‐purine‐2,6‐dione (27) (35 mg, 0.14 mmol, 1 equivalent [equiv]) in dimethylformamide (2 mL) was added potassium carbonate (37.5 mg, 0.27 mmol, 2 equiv) and 2-methoxyethoxymethyl chloride (17 μL, 0.15 mmol, 1.1 equiv). After being stirred for 2 h at ambient temperature, the reaction mixture was treated with H_2_O and directly purified via high-performance liquid chromatography (HPLC) (X-bridgeC18, MeCN/H_2_O/NH_3_) to afford Cpd **3** (33 mg) in 80 to 90% liquid chromatography mass spectrometry purity. Further purification via HPLC (SunfireC18, MeCN/H_2_O/TFA) afforded pure Cpd **3** as colorless wax (10.3 mg, 22%). ^1^H NMR (400 MHz, d^6^-DMSO) δ 6.03 (s, 2H), 3.71 to 3.67 (m, 2H), 3.50 (s, 3H), 3.46 (s, 3H), 3.43 to 3.39 (m, 2H), 3.27 (s, 3H), 3.18 (s, 3H); mass spectrometry (positive electrospray ionization): *m/z* calculated for C_12_H_19_N_4_O_5_S [M+H]^+^ 347.1020, found 347.040.

### Protein Expression.

Protein expression for the ^13^C,^15^N isotope-labeled MLKL N-terminal executioner domain (residues 2 to 154) was done in Minimal Medium containing ^13^C glucose and ^15^N ammonium chloride by Trenzyme GmbH as a glutathione-*S*-transferase (GST) fusion protein including a thrombin cleavage site. After cell lysis by sonication in buffer A [50 mM Tris, 250 mM NaCl, 2.5 mM CaCl_2_, 10% glycerol, 0.1% Tween 80, and 0.5 mM Tris(2-carboxyethyl)phosphine (TCEP), pH 7.5] containing 1 complete protease inhibitor tablet (Roche catalog no. 11836145001) and 1 µg of DNase (Roche catalog no. 10104159001) per 50 mL of buffer, the lysate was centrifuged for 1 h at 50,000 × *g*. The supernatant was loaded to a glutathione Sepharose column (GE catalog no. 17-5131-02) equilibrated with buffer A. The loaded column was washed five times with 30 mL of buffer A and then eluted with 10 times 10 mL of buffer A containing additionally 30 mM reduced glutathione. After cleaving off the fusion protein with bovine thrombin (Sigma catalog no. T4648 100KU) in 20 mM Tris (pH 7.5) containing 250 mM NaCl and 2 mM TCEP overnight at 4 °C, 50 µg/mL Pefabloc was added. GST and MLKL were separated on a size-exclusion chromatography (GE Superdex 75, 26/600 column) in 20 mM Na_2_HPO_4_ (pH 7.5) containing 150 mM NaCl and 5 mM dithiothreitol (DTT). Protein purity was checked by sodium dodecyl sulfate/polyacrylamide gel electrophoresis. The expression of the MLKL executioner domain for X-ray crystallography (residues 2 to 150) was performed in Luria–Bertani medium and purified analogously as a GST fusion protein with the thrombin cleavage site.

### X-Ray Crystallography.

The MLKL executioner domain (residues 2 to 150) crystallized by the hanging-drop vapor-diffusion method with a reservoir solution of 31% polyethylene glycol monomethyl ether 2,000, 0.15 M potassium bromide, and 0.1 M Tris (pH 9). A 20.5 mg/mL protein solution was mixed at a ratio of 1:1 with the reservoir solution and incubated at 4 °C. Cocrystallization was performed with a precipitant solution containing additionally a concentration of 2 mM of the compound. Crystals were obtained with a 1:1 mixture of the protein solution and a reservoir solution containing 32% polyethylene glycol monomethyl ether 2,000, 0.15 M potassium bromide, and 0.1 M Tris (pH 9).

Crystals were frozen in liquid nitrogen directly from mother liquor. Data were collected at the Swiss Light Source (SLS) beamline X10SA (Paul Scherrer Institute) using the PILATUS 6M-F detector. MLKL executioner domain crystals belonged to space group P2_1_ and contained three monomers per asymmetric unit. Liganded crystals adopted space group P4_3_ with two monomers per asymmetric unit. Due to a strong local symmetry between the two molecules in the asymmetric unit, diffraction data merging in 4/mmm resulted in very good statistics as well. Images were processed with XDS (28) and autoPROC (29). The resolution limits were set by using default autoPROC settings. The structures were solved by molecular replacement using MOLREP (30), Phaser (31), DIMPLE (32), and Protein Data Bank (PDB) ID code 4BTF (8) or the unliganded structure as a start model. Subsequent model building and refinement was done by using standard protocols using CCP4 (32) programs, Coot (33), Phenix (34), and autoBuster (35). A summary of the statistics can be found in *SI Appendix*, Table S2. Figures showing structures were made with the PyMOl software.

### Structure Determination by NMR Spectroscopy.

All NOESY experiments for structure calculation were recorded on a Bruker Avance III 800-MHz NMR spectrometer equipped with a TCI cryogenic probe in 2.5-mm NMR tubes at 298 K. Assignment experiments were either recorded on the same spectrometer or on a Avance III HD 600-MHz NMR spectrometer equipped with a QCI cryogenic probe. Two samples were necessary for each of the structure calculations of the MLKL N-terminal executioner domain (residues 1 to 154): one in a 20 mM sodium phosphate buffer at pH 7.5 containing 150 mM sodium chloride and 5 mM deuterated DTT and one in the same buffer, but with D_2_O instead of H_2_O and an adjusted pH of 7.1 to compensate for the isotope effect of deuterium. For the MLKL N-terminal executioner domain, unliganded structure protein concentrations of 430 µM (D_2_O buffer) and 400 µM (H_2_O buffer) were used. In the case of the MLKL:Cpd **3** complex, 340 µM (H_2_O buffer) and 385 µM (D_2_O buffer) protein and 1,200 µM and 840 µM Cpd **3** were mixed. It was checked with ^1^H,^13^C-correlation spectra if MLKL was completely occupied with Cpd **3**. For the assignment of the backbone and side-chain resonances of the MLKL N-terminal executioner domain, the published assignments from Su et al. (11) (Biological Magnetic Resonance Data Bank code 25135) were used as a starting point. Assignments were confirmed for the unliganded protein and transferred to the MLKL:Cpd **3** complex by using triple-resonance experiments (HNCACB, CBCACONH, HNccH total correlated spectroscopy [TOCSY], HNCC TOCSY, HCCh TOCSY, and HcCH TOCSY) (36). Chemical-shift referencing was done directly for ^1^H and indirectly for ^13^C and ^15^N by using the methyl group signals of sodium trimethylsilylpropanesulfonate (37). Distance restraints were derived from ^15^N-edited NOESY (H_2_O buffer) and two separate ^13^C-edited NOESY spectra (D_2_O buffer) for the aliphatic and the aromatic carbon-bound protons. The NOESY mixing time was set to 120 ms. For the complex, an additional two filtered ^13^C-edited NOESY spectra (38) separate for the aliphatic and aromatic carbon-bound protons were recorded with a mixing time of 200 ms to identify intermolecular NOEs. The spectra were processed by using the software Topspin 3.5 (Bruker Biospin) and analyzed by using CcpNmr analysis (39). Torsion angle restraints were calculated from chemical shifts by the software TALOS (40). The structure calculation was performed with the software Cyana (41). The error margins for the proton chemical shifts were set to 0.05 parts per million (ppm) and for the heteronuclear chemical shifts to 0.5 ppm. In the final structure calculation, 100 structures were calculated, from which the 20 lowest-energy structures were selected for the NMR structure bundle. Figures showing structures were made with the PyMOl software.

### NMR Relaxation Experiments.

All relaxation experiments were recorded on Bruker Avance III HD 600-MHz NMR spectrometer equipped with a QCI cryogenic probe in 2.5-mm NMR tubes at 298 K. Either 300 µM WT MLKL or 150 µM F148A-mutated MLKL was used in a 20 mM sodium phosphate buffer at pH 7.5 containing 150 mM sodium chloride and 5 mM DTT. The WT protein was measured unliganded, in the presence of 1.2 mM Cpd **3** and in the presence of 12 mM NM and 500 µM IP6. For determination of *T*_1_, 10 relaxation delays, including two duplicates between 10 ms and 2 s, were recorded. Each spectrum was recorded with 128 complex points in the indirect dimension, an interscan delay of 2 s, and eight scans. For *T*_1ρ_, eight relaxation delays, including two duplicates between 4 and 96 ms, were recorded. For the WT protein in the presence of NM and IP6, an additional relaxation delay of 128 ms was recorded. Each spectrum was recorded with 128 complex points in the indirect dimension, an interscan delay of 2 s, and 16 scans. Exchange-free *T*_2_ time constants were calculated from *T*_1_ and *T*_1ρ_ time constants. The heteronuclear NOE was recorded in an interleaved experiment with 128 complex points per spectrum, an interscan delay of 2 s, and 16 scans. For the F148A sample, all experiments were run with doubled scans. Peak integration for nonoverlapping peaks and fitting of relaxation data were done with CcpNmr analysis (39). The fitting error is reported in the error bars for *T*_1_. For *T*_2_ and τ_C_, error propagation based on the fitting error of *T*_1_ and *T*_1ρ_ was performed.

### Functional NMR Assay.

For the functional NMR assay ^1^H,^15^N-correlation spectra were recorded on a Bruker Avance III HD 600-MHz NMR spectrometer equipped with a cryogenic QCI probe in 2.5-mm NMR tubes at 298 K. Spectra were recorded with 64 complex points in the indirect dimension, 96 scans, and an interscan delay of 2 s. For the samples, 80 µM MLKL N-terminal executioner domain (residues 1 to 154) was used in a 20 mM sodium phosphate buffer at pH 7.5 containing 150 mM sodium chloride and 1 mM TCEP. For the different conditions, either 500 µM IP6, 12 mM NM, or a combination of the two were used. The xanthine compounds were tested at 500 µM, while NSA, due to limited solubility, was tested only at 200 µM, which is sufficient to saturate MLKL. Spectra were analyzed, and peaks were integrated in CcpNnmr analysis (39). The population of the α-helix 6 attached state was calculated by using the integral of Trp133 Hε1,Nε1 correlation, which showed distinct chemical shifts in the α-helix 6 attached (inactive) and detached (active) states. In some cases, the Trp133 peak in the inactive state overlapped with the peak of the Hε1,Nε1 correlation of Trp109; in this case, the intensity of the Trp133 Hε1,Nε1 cross-peak was assumed to be half of the overlapped peak.

### NMR CSP Calculation.

CSPs were calculated by using Eq. **1**

$$CSP=\sqrt{{\left(\delta_{H}-\delta_{Href}\right)}^{2}+\frac{{\left(\delta_{N}-\delta_{Nref}\right)}^{2}}{100}},$$ [1]

where δ is the chemical shift of the spectrum in the reference state (ref) or in the compared state in the proton (H) and nitrogen (N) dimension.

### Real-Time NMR.

For the real-time NMR measurements, 300 µM MLKL (1 to 154) in 20 mM sodium phosphate buffer at pH 7.5 containing 150 mM sodium chloride and 1 mM TCEP was used to record a series of ^1^H,^15^N-correlation spectra with a measurement time of 20 min each for 2 d. Spectra were processed by using Topspin 3.5, and peaks were integrated by using CcpNmr analysis (39). The peak intensity of selected peaks was plotted against the elapsed time, and the time curve was then fitted with Eq. **2**.

y=A⋅exp(−B⋅x)+C⋅exp(−D⋅x)+E, [2]

where *B* and *D* are the rates of the biexponential decay, and time constants *T*_*f*_ and *T*_*s*_ were derived by calculating the reciprocal value 1/*B* and 1/*D*, respectively.

### NMR Diffusion.

NMR diffusion experiments were recorded as pseudo two-dimensional experiments with varying gradient strength either as a ^15^N-filtered NMR experiment (42) (recorded with ^15^N-labeled protein). For ^1^H–one-dimensional (^1^H-1D) diffusion experiments (using unlabeled protein), a stimulated echo sequence with a longitudinal eddy current delay was employed using bipolar gradient pulses for diffusion (43) was employed. Water suppression was achieved by a WATERGATE w5 pulse scheme (44). The diffusion time was 80 ms, except for the regular ^1^H-1D experiment for the condition with concentrations of 12 mM and 500 µM without compound, where 200 ms were used, to determine the significantly smaller diffusion constant and, therefore, larger hydrodynamic radius more accurately. In either case, 300 µM MLKL (1 to 154) in 20 mM sodium phosphate buffer at pH 7.5 containing 150 mM sodium chloride and 1 mM TCEP was used. For the ^1^H-1D experiments, dioxane was added as an internal standard to calculate the hydrodynamic radius. For the ^15^N-filtered experiment, the diffusion constant of dioxane from a ^1^H-1D experiment under equivalent conditions was used. Eq. **3** was used for the fitting of a diffusion constant of not-overlapped signals like the protein amide region.

$$y=A\cdot \text{exp}\left(-1\cdot d\cdot x^{2}\right),$$ [3]

where *d* is the diffusion constant. For overlapped peaks, a sum of two or three of the terms in Eq. **2** was used, where the diffusion constants of known components were kept constant. The hydrodynamic radius was calculated by using the determined diffusion constants of the component of interest (*d*) and dioxane (*d*_dioxane_) and the known hydrodynamic radius of dioxan (2.12 Å) (23) using Eq. **4**.

$$r_{H}=2.12\text{Å}\cdot \frac{d_{\mathrm{dioxane}}}{d},$$ [4]

where *r*_*H*_ is the hydrodynamic radius of the component of interest.

### CD Spectroscopy.

CD spectra were recorded on a JASCO J-715 CD spectrometer in a 0.05-cm-path-length cuvette. A concentration of 0.5 mg/mL ^15^N-labeled MLKL executioner domain was measured in a 20 mM sodium phosphate buffer at pH 7.5 containing 150 mM sodium chloride and 0.5 mM TCEP immediately after mixing and in the presence of 12 mM NM and 500 µM IP6 after an incubation period of 4 d. After CD measurement, the activation state of MLKL was confirmed by recording a ^1^H,^15^N-correlation NMR spectrum.

## Supplementary Material

Supplementary File

## Data Availability

Structural information data have been deposited in the PDB (PDB ID codes 6ZVO, 6ZZ1, 6ZPR, and 6ZLE). We plan to provide Cpd **3** as a tool compound free of charge as BI-8925 in the course of the Boehringer Ingelheim Open Innovation initiative (https://opnme.com/molecules/mlkl-bi-8925).
