# Supplementary material for: Locking mixed-lineage kinase domain-like protein in its auto-inhibited state prevents necroptosis
Source: Proc Natl Acad Sci U S A. 2020 Dec 14;117(52):33272–81. doi: 10.1073/pnas.2017406117 (PMC7777204; doi:10.1073/pnas.2017406117)
Supplement: Supplementary File [file pnas.2017406117.sapp.pdf]

Supplemental Figures

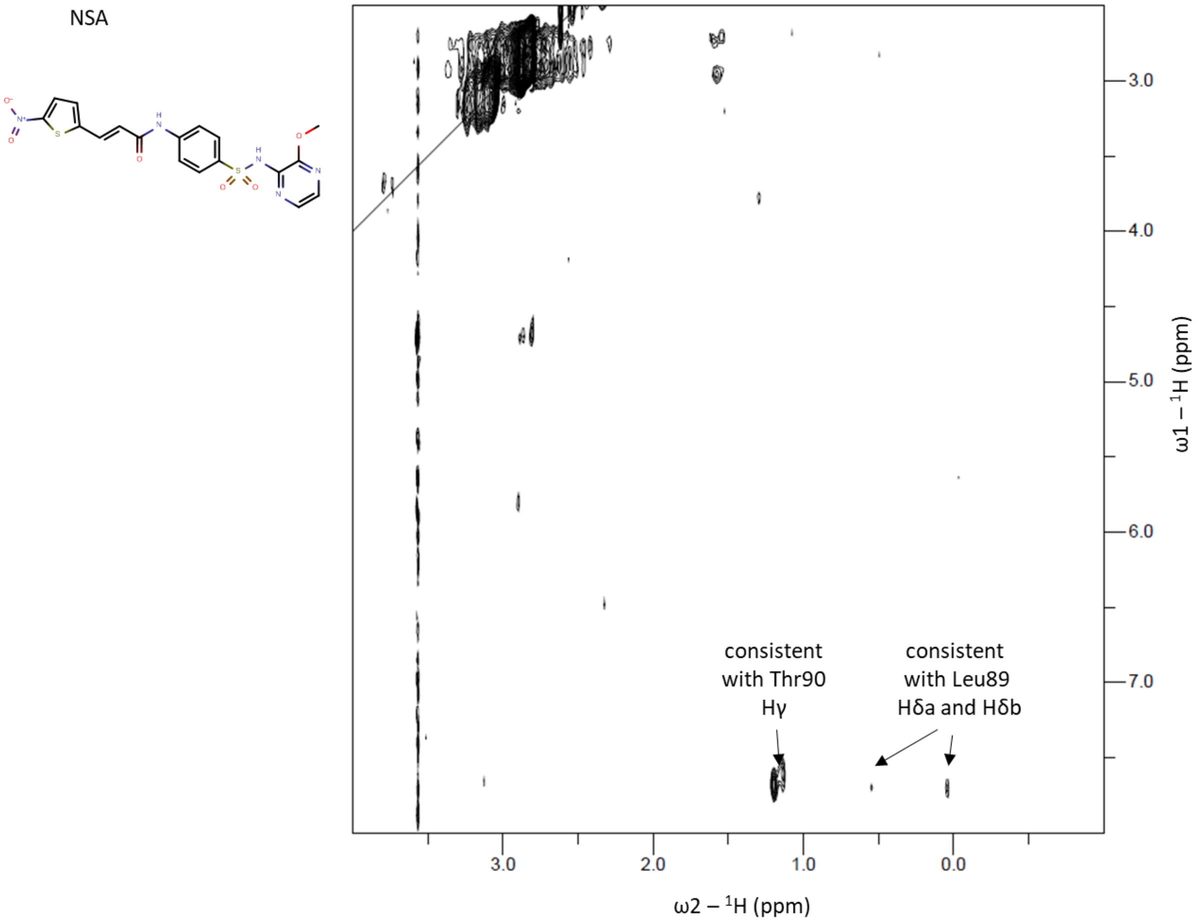

Figure S1: Projection of 3D ^{13}C-edited-filtered NOESY spectra of the MLKL executioner domain in complex with NSA.

www.pnas.org/cgi/doi/10.1073/pnas.2017406117

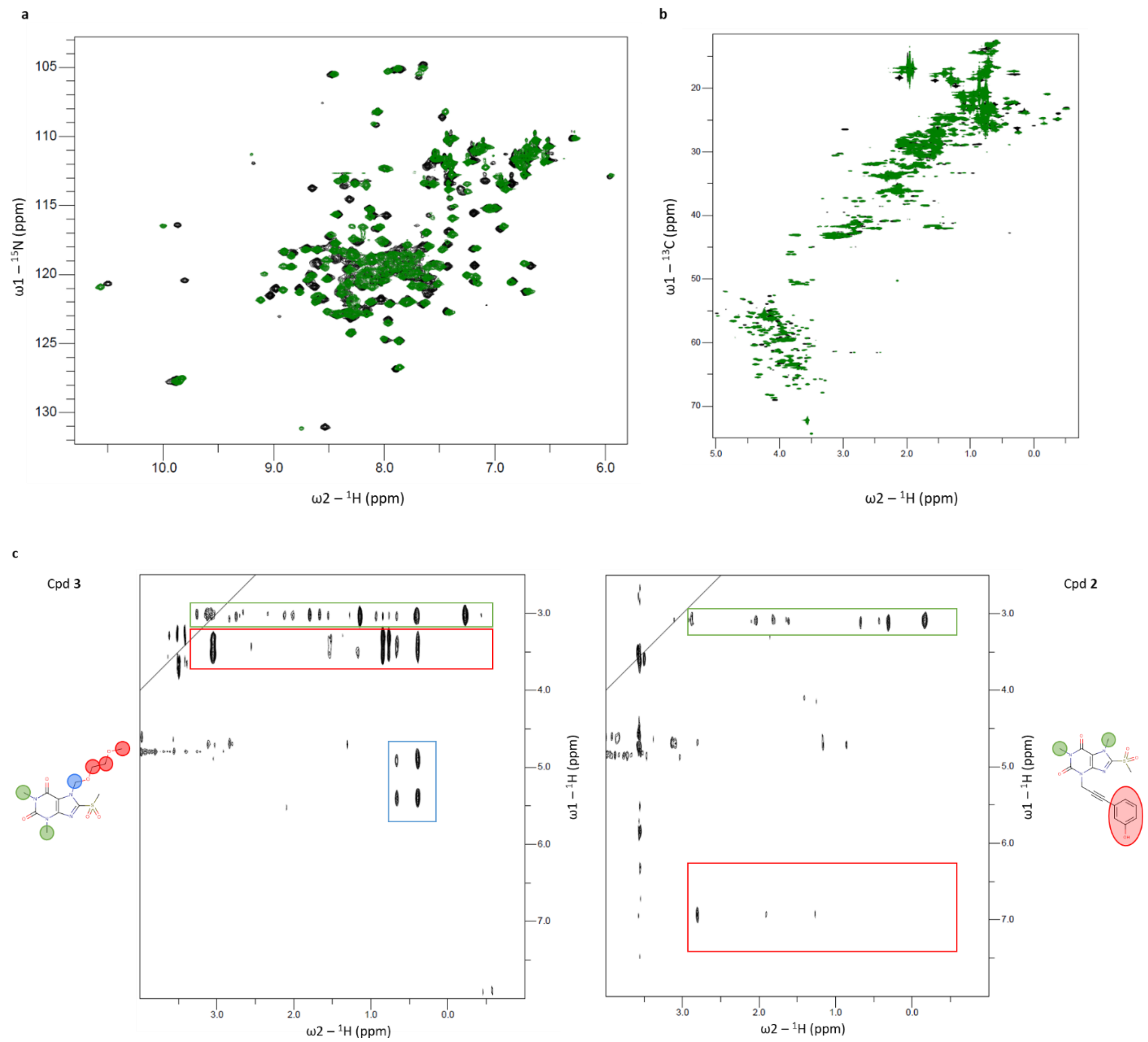

*Figure S2: $^{1}H,^{15}N$ (a) and $^{1}H,^{13}C$ (b) correlation spectrum of the unliganded MLKL executioner domain (black) and in complex with Cpd **2** (green). c: Projections of 3D ^{13}C-edited filtered NOESY spectra of the MLKL executioner domain in complex with Cpd **3** (left) and Cpd **2** (right). Proton positions in the structural formulas of the compounds and corresponding strips in the NOESY spectra are color-coded.*

a b c

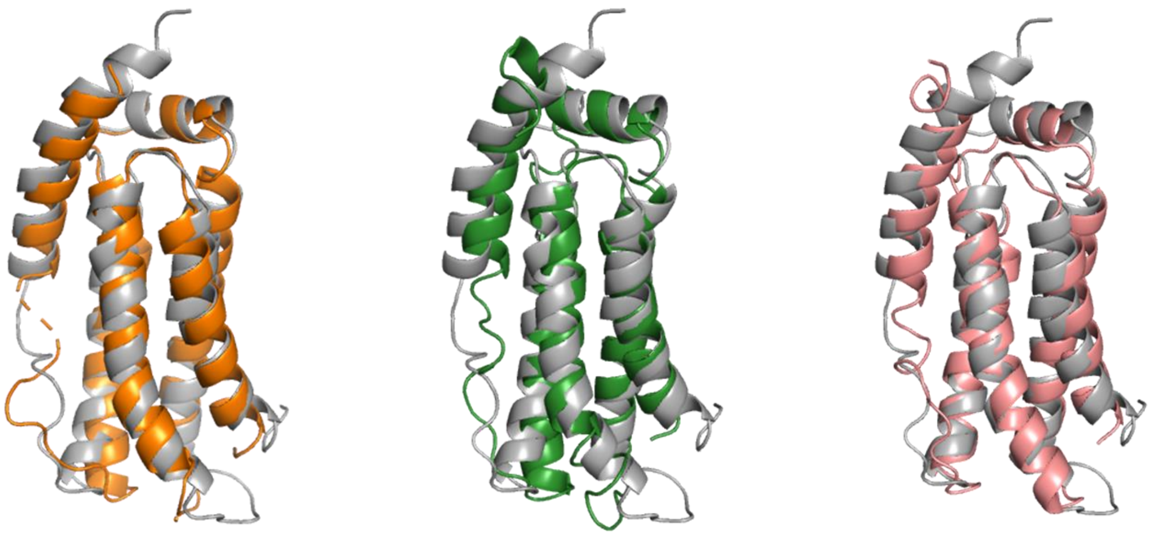

r.m.s.d.: 2.1 Å r.m.s.d.: 2.4 Å r.m.s.d.: 2.5 Å

Figure S3: Superimposition of unliganded MLKL executioner domain NMR structure (grey, PDB: 6ZLE) with the X-ray structure (a; orange, PDB: 6ZVO) and the published NMR structures with the PDB codes 2MSV[11] (b; green) and 6D74[12] (c; salmon). Below the r.m.s.d. values between the structures are indicated. R.m.s.d. values were determined by the protein structure alignment tool of Maestro.

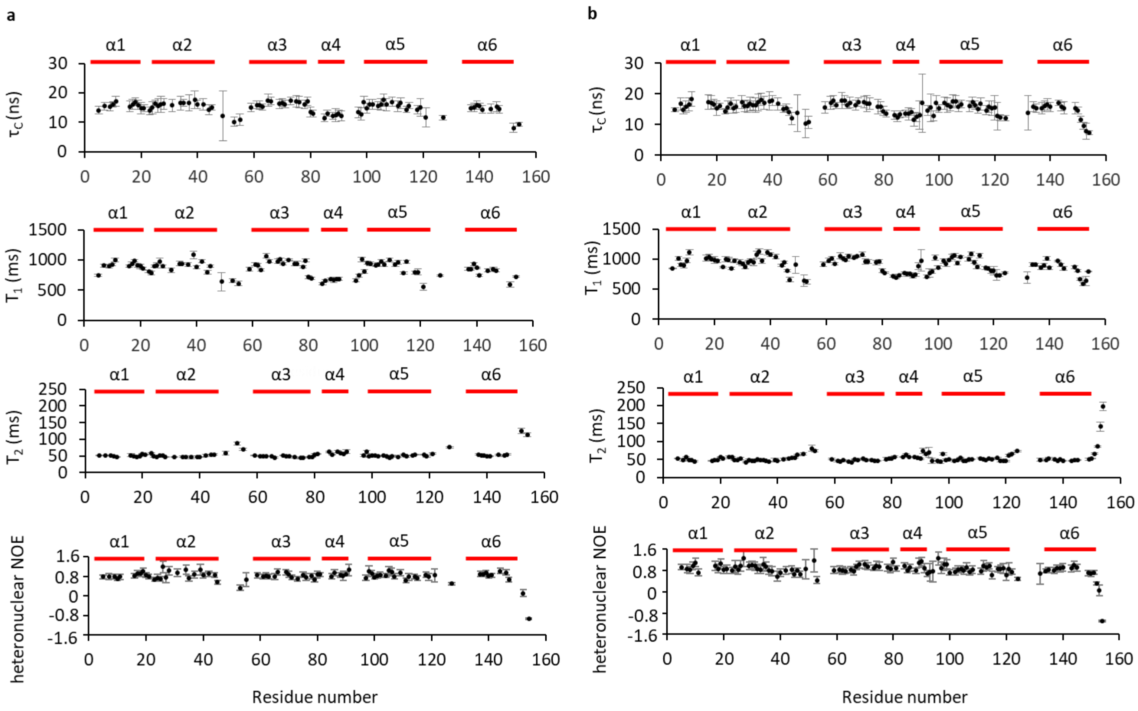

*Figure S4: ^{15}N NMR relaxation data for unliganded MLKL executioner domain (a) and in complex with Cpd **3** (b). Only non-overlapping cross peaks were analyzed.*

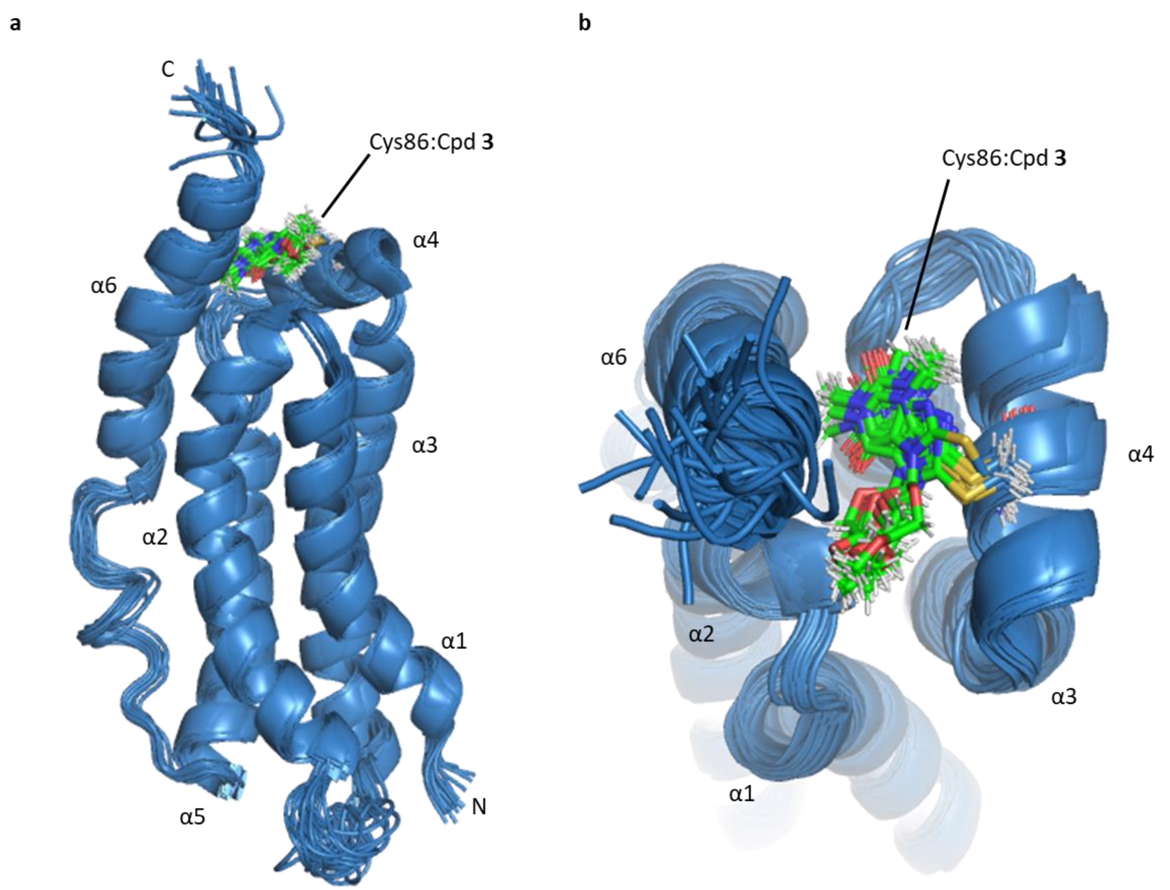

*Figure S5: a: Bundle depicting the 20 lowest energy NMR structures of the MLKL executioner domain in complex with Cpd **3**. b: Close up of the covalently attached Cpd **3** (green colored sticks) highlighting the well defined binding mode.*

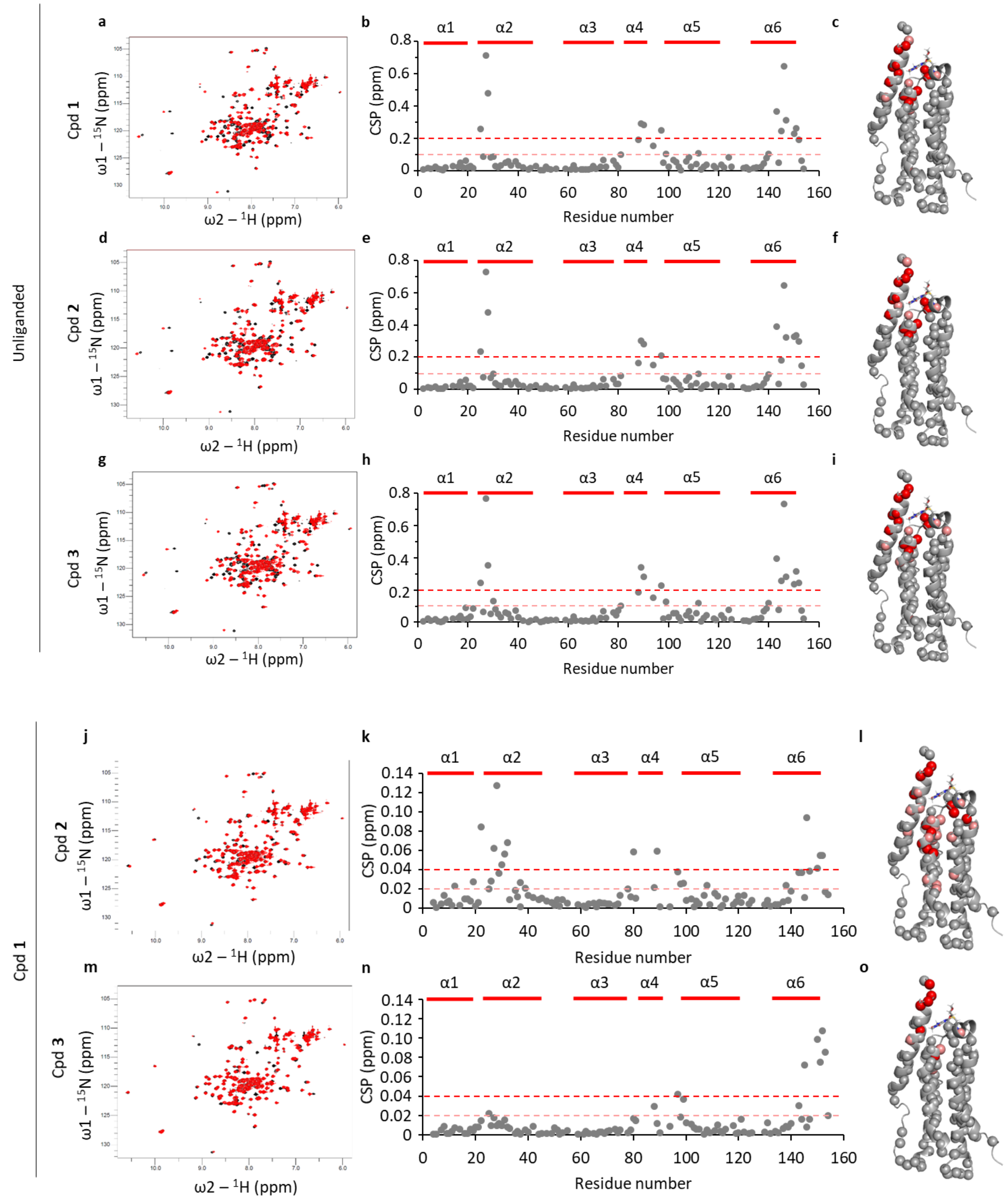

*Figure S6: Depicted are superimpositions of ^{1}H,^{15}N correlation spectra of unliganded MLKL executioner domain in black and in complex with Cpd **1** (a), Cpd **2** (d) or Cpd **3** (g) in red. In the lower half of the figure ^{1}H,^{15}N correlation spectra of the MLKL executioner domain:Cpd **1** complex are depicted in black and in complex with Cpd **2** (j) and Cpd **3** (m) in red. Next to the spectra corresponding CSP plots between the two spectra are shown (b, e, h, k, n). On the right CSP larger than 0.1 ppm are plotted in salmon and larger than 0.2 ppm in red on the NMR co-structure of MLKL with Cpd **3** (c, f, i). In case of the comparisons with the Cpd **1** complex CSP larger 0.02 ppm are indicated by salmon colored spheres and larger than 0.04 ppm by red colored spheres (l, o).*

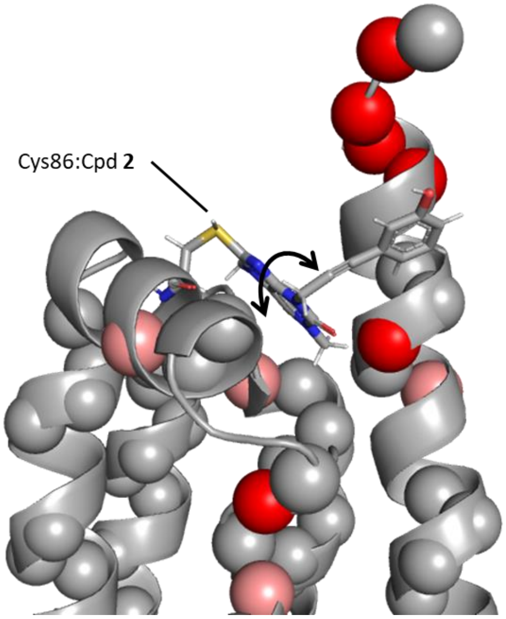

*Figure S7: Modelled binding pose of Cpd **2** to the MLKL executioner domain created by the software Maestro. Salmon spheres indicate residues experiencing changes in chemical shift larger than 0.02 ppm and red spheres larger than 0.04 ppm between ^{1}H,^{15}N correlation spectra of MLKL bound to Cpd **1** or Cpd **2**. The black arrow indicates that the side chain of the compound is able to rotate freely around the indicated bond.*

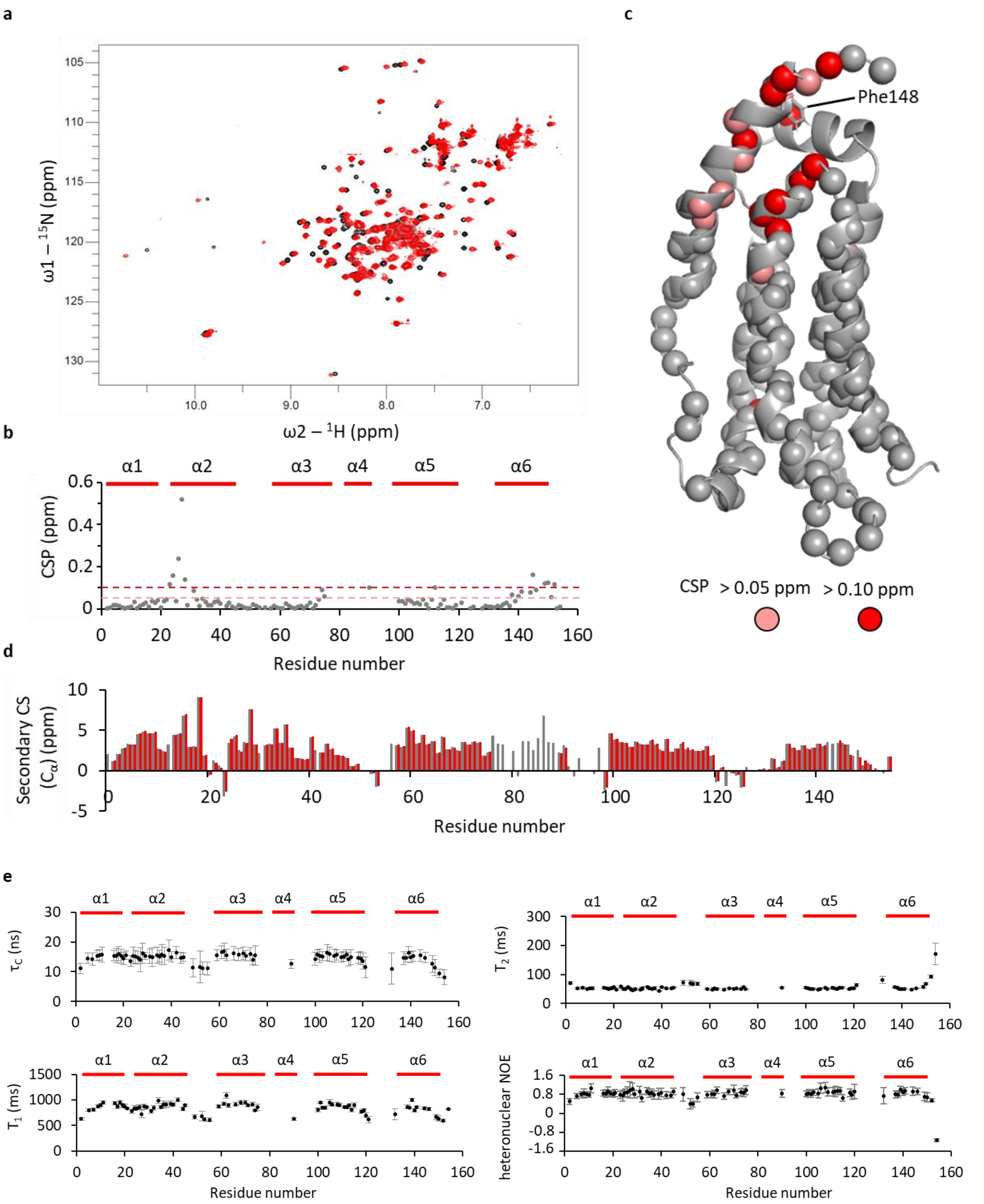

Figure S8: a: $^{1}H,^{15}N$ correlation spectrum of the wild-type MLKL executioner domain (black) and the F148A mutated variant (red). b: Chemical shift perturbation (CSP) extracted from the two spectra depicted in a plotted against the primary sequence of the protein. c: Spheres on the NMR structure indicate residues for which CSPs could be evaluated. Red spheres indicate CSPs larger 0.1 ppm, while salmon spheres indicate CSPs between 0.05 and 0.1 ppm. d: Secondary chemical shifts of Cα atoms of the wt protein (grey) and the F148A mutated variant (red). e: ^{15}N relaxation parameters of the F148A mutated variant. Only non-overlapping cross peaks were analyzed. Residues comprising α-helix 4 in wild-type MLKL could not be assigned in MLKL F148A due to line broadening. Therefore, these residues could not analyzed in b-e.

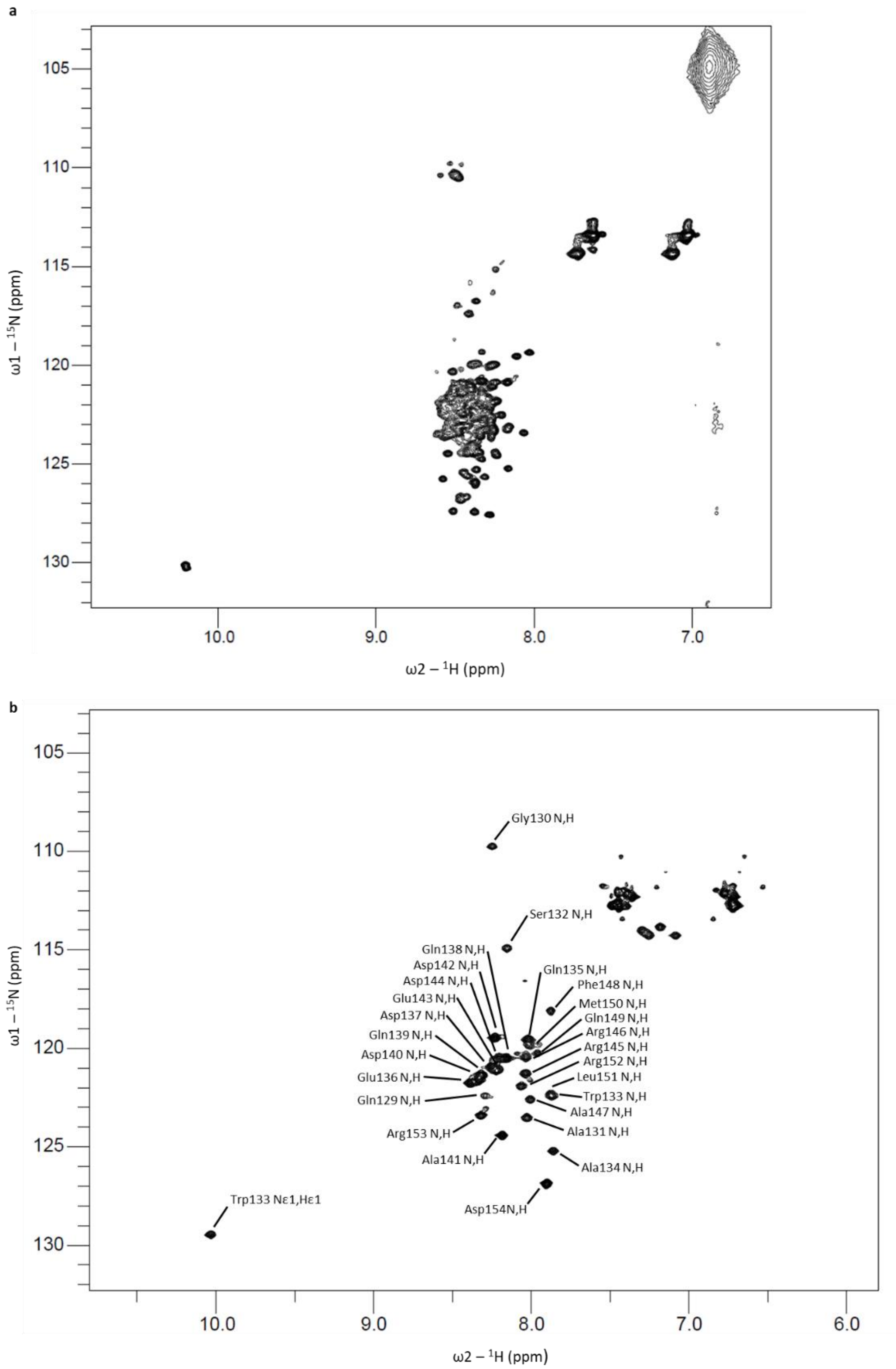

Figure S9: a: $^{1}H,^{15}N$ correlation spectrum of the MLKL executioner domain unfolded by 5.7 M guanidinium chloride. A very narrow ^{1}H dispersion typically for fully unfolded protein is observed. b: Backbone assignment of the MLKL executioner domain in the active state induced by the presence of 12 mM NM and 200 µM IP6 after an incubation of 3 days.

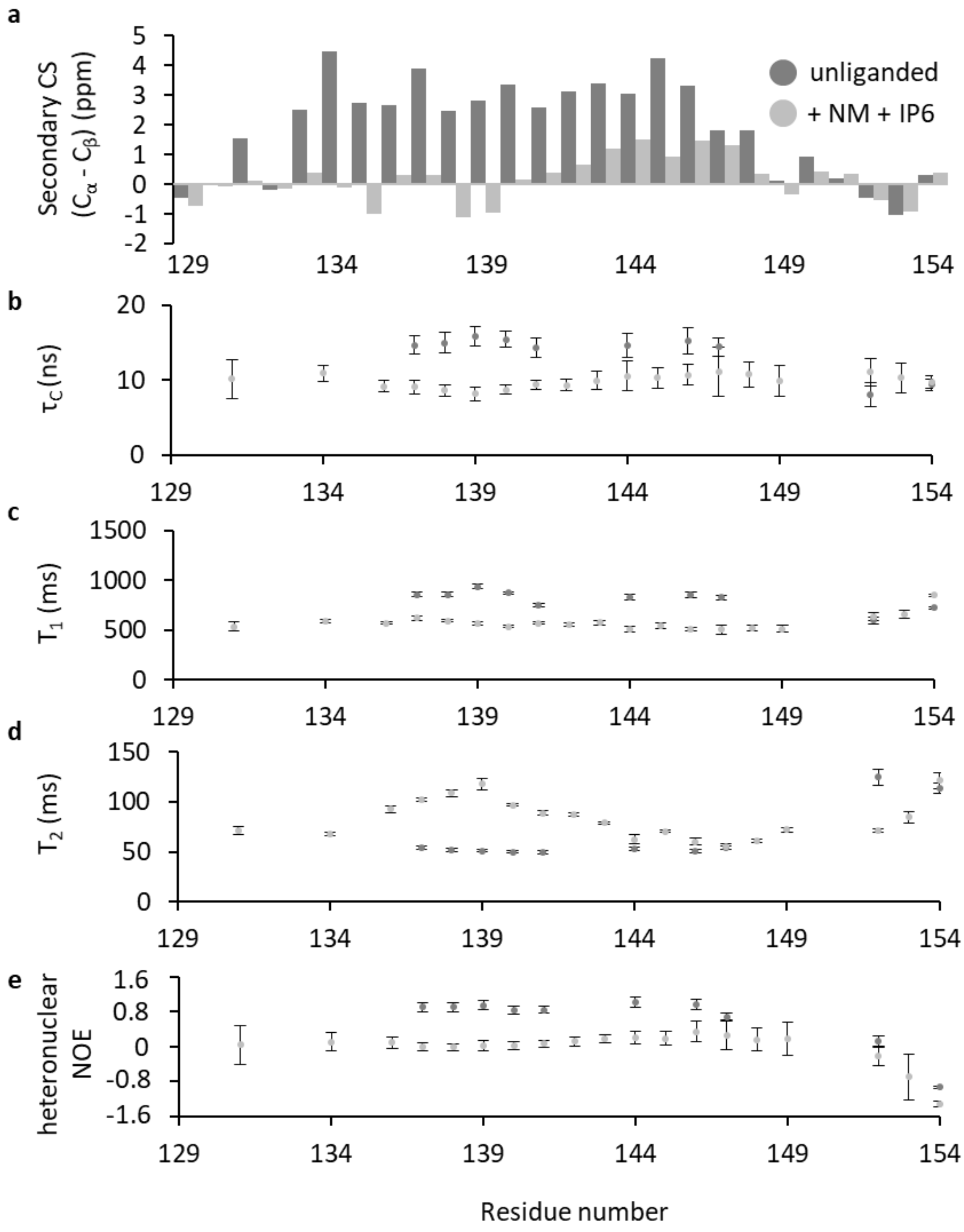

Figure S10: Secondary chemical shifts (a) , ^{15}N-Relaxation data (b-d) and heteronuclear NOE values (e) of visible peaks in the active state of the MLKL executioner domain induced by the presence of NM and IP6 (light grey) compared to the monomeric unliganded protein (dark grey). For the relaxation parameters only non-overlapping peaks were analyzed.

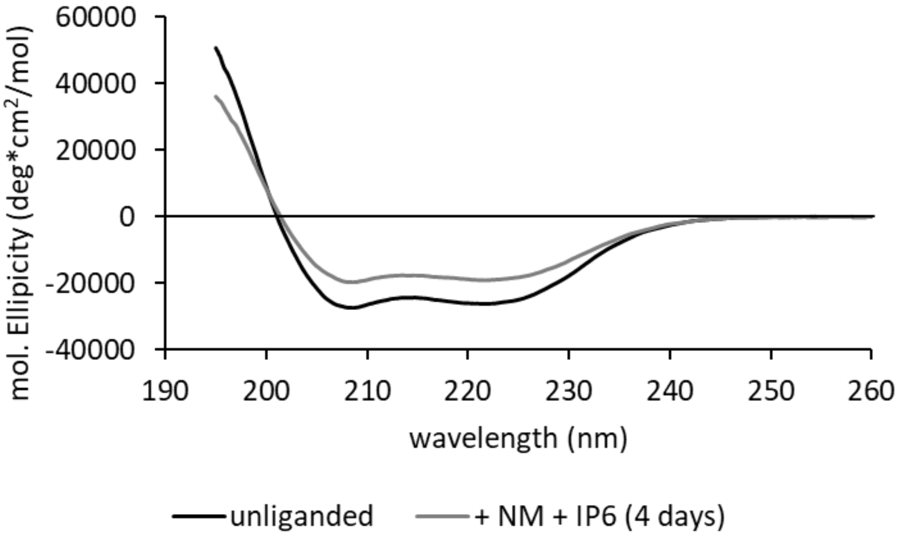

Figure S11: CD spectra of MLKL unliganded (black) and in presence of 12 mM NM and 500 µM IP6 after 4 days of incubation (light grey)

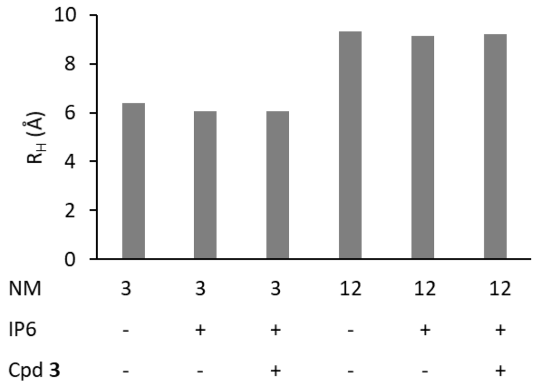

Figure S12: Hydrodynamic radii of NM extracted from NMR diffusion experiments at different concentrations (indicated below plot in mM) in presence or absence of 500 µM IP6 and/or 500 µM Cpd3 (presence indicated by plus and minus signs, respectively).

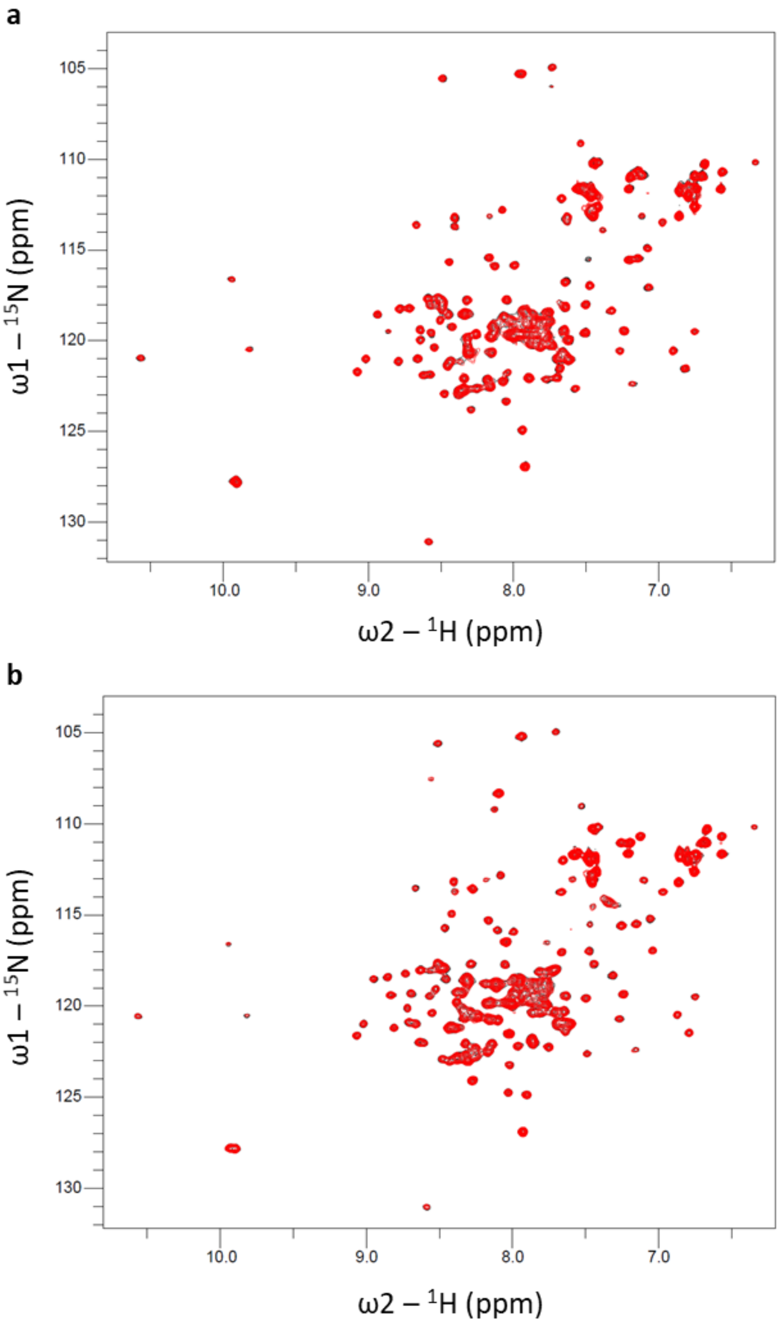

Figure S13: a: $^{1}H,^{15}N$ correlation spectrum of the MLKL executioner domain recorded directly after the addition of 12 mM NM (black) and after 87 h of incubation at 298 K (red). b: $^{1}H,^{15}N$ correlation spectrum of the MLKL executioner domain recorded directly after the addition of 500 µM IP6 (black) and after 66 h of incubation at 298 K (red).

Supplemental Tables

Table S1: Statistics of NMR structures

	**Unliganded**<br>6ZLE	**Cpd 3 complex**<br>6ZPR
Resonance assignment*	95.1 %	95.6 %
NOE cross peaks	4209	4594
assigned	3795	4029
unassigned	414	525
Intermolecular NOEs	-/-	41
fulfilled	-/-	37
violated	-/-	4**
Upper distance limits	2798	2925
short-range ($\vert i-j\vert \leq 1$)	1347	1396
medium-range ($1 < \vert i-j\vert < 5$)	783	838
long-range ($\vert i-j\vert \geq 5$)	668	691
Ramachandran*		
most favored	87.7 %	87.7 %
additionally allowed	11.8 %	11.3 %
generously allowed	0.3 %	0.8 %
disallowed	0.3 %	0.1 %

*from Cyana software **violated on average by 1.40 Å

Table S2: Data collection and refinement statistics (molecular replacement)

	**Unliganded*** 6ZVO	**Cpd 3 complex*** 6ZZ1
**Data collection**		
Space group	$P2_1$	$P4_3$
Cell dimensions		
a, *b*, *c* (Å)	56.057, 53.501, 70.912	56.709, 56.709, 90.501
α, β, γ (°)	90.0, 98.363, 90.0	90.0, 90.0, 90.0
Resolution (Å)	1.375-70.158 (1.375-1.483) **	1.640-19.705 (1.640-1.746)
R_{merge}	0.045 (1.096)	0.027 (0.714)
$I / \sigma I$	18.3 (1.4)	24.6 (1.4)
Completeness sp / el (%)	76.3 / 94.4 (18.8 / 59.6)	82.8 / 94.4 (24.3 / 55.7)
Redundancy	6.5 (6.6)	6.7 (5.5)
**Refinement**		
Resolution (Å)	1.371-70.16	1.640-20.05
No. reflections	65995	28985
R_{work} / R_{free}	0.1896 / 0.2335	0.1966 / 0.2311
No. atoms		
Protein	3694	2340
Ligand/ion	4	38
Water	450	154
B-factors		
Protein	26.0	51.8
Ligand/ion	32.1	64.6
Water	36.1	53.4
R.m.s. deviations		
Bond lengths (Å)	0.010	0.010
Bond angles (°)	0.94	0.90
Ramachandran		
Favored regions	99.3 %	98.9 %
Allowed regions	0.7 %	1.1 %
Disallowed	0.0 %	0.0 %

*For each structure one crystal has been used. **Values in parentheses are for highest-resolution shell.

Table S3: Time constants for the fast (T_f) and slow (T_s) phase of dispersed peaks in MLKL executioner domain in presence of 12 mM NM and 50 µM IP6 observed by real-time NMR. Peak intensities were fitted by equation 1.

**Residue**	**Secondary structure element**	**T_s (h)**	**ΔT_s (h)**	**T_f (h)**	**ΔT_f (h)**
Thr9	α-helix 1	9.9	0.5	0.37	0.04
Gly11	α-helix 1	8.9	0.5	0.23	0.04
Lys25	α-helix 2	9.4	0.4	0.41	0.04
Leu31	α-helix 2	8.6	0.4	0.24	0.04
Met62	α-helix 3	10.7	0.5	0.40	0.05
Asn73	α-helix 3	10.2	0.5	0.45	0.05
Ile85	α-helix 4	11.2	0.5	0.24	0.08
Thr90	α-helix 4	8.7	0.4	0.40	0.06
Lys99	α-helix 5	11.8	0.7	0.40	0.04
Leu105	α-helix 5	8.8	0.4	0.25	0.04
Trp133 Nε1.Hε1	α-helix 6	14.3	0.5	0.52	0.04
Asp137	α-helix 6	10.1	0.5	0.45	0.05
Gln138	α-helix 6	10.1	0.5	0.35	0.04
Gln139	α-helix 6	10.7	0.5	0.38	0.03
Asp140	α-helix 6	10.0	0.4	0.30	0.03
Ala141	α-helix 6	8.2	0.3	0.22	0.03
Asp144	α-helix 6	9.0	0.4	0.29	0.04
Arg146	α-helix 6	10.2	0.4	0.35	0.04
**Average**		**10.0**		**0.35**	
**Standard deviation**		**1.0**		**0.07**	
